# GTsurvival: A Hybrid GCN-Neural Decision Tree Model for Restricted Mean Survival Time Prediction with Complex Censored Data

**DOI:** 10.3390/e28010028

**Published:** 2025-12-25

**Authors:** Jingyi Zhang, Shishun Zhao, Dongmei Lu, Jianhua Cheng

**Affiliations:** 1 School of Mathematics, Jilin University, Changchun 130012, China; jyzhang23@mails.jlu.edu.cn (J.Z.); zhaoss@jlu.edu.cn (S.Z.); 2 National Applied Mathematical Center (Jilin), Changchun 130012, China; 3 Department of General Education, Changchun College of Electronic Technology, Changchun 130012, China

**Keywords:** survival analysis, graph convolution network, neural decision tree, restricted mean survival time

## Abstract

Chronic diseases, particularly those with progressive neurological impairment, present a significant challenge in healthcare due to their impact on millions globally and the limited availability of effective therapies. Addressing this challenge requires innovative approaches, such as leveraging individuals’ genetic features for early intervention and treatment strategies. Due to the irregular intervals of patient visits, clinical data typically appear as censored, necessitating advanced analytical methods. Thus, this study introduces GTsurvival, a novel network architecture that combines graph convolutional networks (GCN) with a neural decision tree, providing promising advancements in disease prediction. GTsurvival utilizes restricted mean survival time (RMST) as pseudo-observations and directly connects them with baseline variables. Through the joint simulation of RMST, GTsurvival can effectively utilize shared information and enhance its predictive ability for patients’ future survival status. Firstly, GTsurvival is introduced to handle complex censored data, emphasizing the crucial role of graphs utilized in GCNs for processing related information among samples. Secondly, the neural decision tree within GTsurvival enhances decision-making by mitigating uncertainty at split nodes, effectively minimizing the global loss function and optimizing survival analysis in high-dimensional datasets. Thirdly, evaluations on simulated datasets and a real-world neurodegenerative disease cohort verify that the proposed GTsurvival method surpasses existing approaches. This superiority is partly attributed to the inclusion of a generalized score test during feature selection, which helps capture variants associated with disease progression.

## 1. Introduction

Chronic progressive diseases, particularly those affecting neurological function, present significant challenges to global healthcare systems. These conditions often involve complex interactions between genetic predisposition and environmental factors, leading to gradual decline in patient health. According to the World Health Organization [1], approximately 55 million people are currently suffering from Alzheimer’s disease (AD), with projections indicating a significant rise to 78 million by 2030 and 139 million by 2050. Due to the limited effectiveness of disease treatments, early prediction plays a crucial role in enhancing the quality of life for patients during disease advancement. Notably, genetic-based predictive models have recently garnered considerable attention. Existing models predominantly rely on polygenic risk scores [2] (PRSs) to handle genetic variations; however, these scores are essentially weighted linear sums of genetic factors. Despite their ease of implementation, PRSs ignore complex nonlinear interactions among high-dimensional genetic variants. The methods cannot automatically handle complex relationships among high-dimensional covariates, for instance, nonlinear associations between covariates and outcomes, or interplays among covariates. Furthermore, due to intermittent subject examinations, it was not feasible to observe the precise time of disease onset, posing a challenge for the analysis of the longitudinal cohort data [3]. These limitations mentioned above, particularly in modeling nonlinear genetic interactions and handling censored data, highlight the need for prediction models that can address both high-dimensional nonlinearity and censoring mechanisms.

To address these challenges, we propose integrating restricted mean survival time [4] (RMST) analysis with nonlinear machine learning algorithms. RMST has received widespread attention in the fields of medicine and biology owing to its direct and effective clinical interpretability while circumventing strong parametric assumptions required by traditional survival metrics. Specifically, RMST calculates the average survival time up to a specific observation time point through numerical integration of the survival curve, making it particularly suitable for handling the censored data. Statistical approaches based on Cox regression have demonstrated efficacy in analyzing RMST within simple covariate settings, as evidenced by applications in conditions like acute coronary syndrome [5] and chronic liver disease [6]. Recent pseudo-observation methods [7] have been extended to RMST analysis with time-varying covariates through generalized linear models, yet these methods remain limited by the requirement for explicit functional structures when utilized in high-dimensional genomic studies. Bouaziz (2023) [8] developed asymptotic formulas to accelerate computations for RMST in the context of right-censored and interval-censored data.

Deep learning offers novel approaches for high-dimensional biological data analysis, using flexible architectures to overcome traditional modeling constraints. Notable developments include Zhao’s [9] multi-timepoint RMST prediction framework using inverse probability of censoring weighting, and Sun and Ding’s [10] neural network for interval-censored survival analysis and identification of subgroups with differential progression risks. However, these approaches exhibit limited capacity for processing non-Euclidean data structures inherent to biological systems, such as cellular interaction graphs. The foundational work of Kipf and Welling [11] established graph convolutional networks (GCNs) as a framework for handling graph-structured data, with subsequent biological applications by Peng et al. [12] in cellular classification modeling and Xu et al. [13] in cell graph construction. Further advancements, such as FGCNSurv [14], employ dual-fused convolutional architectures for multi-omics survival prediction, and Ling et al. [15] developed a sparse geometric graph approach with sequential feature selection. While existing GCN-based survival models have shown progress, their implementations primarily focus on right-censored data. Based on the research mentioned above, we may face two problems: (1) progression data for many chronic conditions often exhibit complex censored types (e.g., interval censoring from irregular clinical visits combined with right censoring), which existing GCN architectures might not fully accommodate, and (2) conventional survival frameworks might inadequately capture nonlinear genetic interactions. These limitations could restrict the clinical translation of graph-based models for disease prediction.

To overcome these problems, our work includes the key components: (1) Loss function for complex censoring: we utilize a subject-specific mean squared error loss based on RMST pseudo-values, constructed via the Jackknife method. This approach directly models the relationship between covariates and RMST for interval-censored data. It differs from and extends prior works like Zhao et al. [9], which designed loss functions primarily for right-censored data. (2) Graph construction for patient similarity: We construct a patient similarity graph (nodes as patients, edges via KNN based on genomic Euclidean distance) as input to a GCN. This allows the model to leverage relational structure among patients, contrasting with standard neural networks (e.g., Sun et al. [10]) that process covariates independently. (3) Integration of the GCN with the neural decision tree: We combine the GCN for relational feature learning with subsequent neural decision tree for final prediction. This hybrid architecture, which integrates graph-based representation learning with tree-based prediction, is not a feature of existing pure GCN survival models (e.g., Ling et al. [15]). Specifically, the model jointly optimizes parameters of all splitting nodes and predictive values of leaf nodes via global risk minimization, thus ensuring the overall optimality of the model. (4) To enhance interpretability, we combine the generalized score test for identifying disease-associated genetic variants, with post-hoc explanation methods (e.g., LIME [16]) to interpret the importance of input features for individual model predictions. Notably, the GCN module in our model is critical to genomic data processing: by iteratively propagating and aggregating information across the gene network, each node integrates neighbor information. This information propagation and aggregation process inherently reduces feature redundancy (corresponding to entropy reduction in information theory) and strengthens the relevance between genetic features and survival outcomes. Conceptually, this aligns with the network communication paradigm in information theory, which aims to efficiently extract “signals” most predictive of survival outcomes from high-dimensional, complex genomic data while attenuating “noise” (i.e., irrelevant variations). Evaluations on simulated and real-world genomic datasets demonstrate GTsurvival’s superiority over existing survival methods.

The paper is structured as follows: Section 2 introduces GTsurvival, a survival prediction algorithm that merges GCN with network decision trees [17] to effectively handle complex censored data. Section 3 outlines detailed simulation experiments, with numerical results presented in the tabular format. In addition, the performance of GTsurvival is evaluated on a biomedical dataset, and a generalized score test is conducted to identify representative genetic features. Lastly, Section 4 offers general remarks and discusses our findings.

## 2. Methods

### 2.1. Data Description

Herein, a novel approach GTsurvival is utilized to analyze datasets from the Alzheimer’s Disease Neuroimaging Initiative (ADNI, https://adni.loni.usc.edu/ (accessed on 5 March 2025)), incorporating blood-based gene expression profiles generated by Bristol-Myers Squibb laboratories. The present study initially divided participants into three cohorts according to their cognitive status: cognitively normal (CN), mild cognitive impairment (MCI), and AD. AD-related brain changes begin even before MCI onset. Therefore, this investigation extends beyond MCI patients to include the broader CN/MCI population, aiming to facilitate early detection of AD and enhance the quality of life both prior to and throughout disease progression. Considering that the ADNI cohort includes individuals aged 55 years or above, and AD onset seldom occurs before age 55 [18], 55 years is established as the baseline for time-to-event measurement (years until AD diagnosis). Our dataset comprises 744 genomically sequenced participants with censoring distributed as follows: left-censored (*n* = 44), interval-censored (*n* = 192), and right-censored (*n* = 508). Microarray gene expression datasets are available at https://ida.loni.usc.edu/explore/jsp/search/search.jsp?project=ADNI#geneticFiles (accessed on 5 March 2025). The raw gene expression values were pre-processed utilizing the Robust Multi-chip Average (RMA) normalization method, with duplicate genes aggregated by mean expression to yield 20,093 final genetic features.

### 2.2. Interval Censoring

In this study, it is assumed that rather than directly obtaining survival time *T*, a random interval [L,R] (where L≥0 and L≤R) is observed, which almost certainly encloses the event time, as denoted by $P\left(T\in [L,R]\right)=1$ [19]. The right end of the interval can take infinite values, classified as follows:

- If 0<L<R<∞, the data are strictly interval-censoring;
- If 0=L<R<∞, the data are left-censored;
- If 0<L<R=∞, the data are right-censored;
- If 0<L=R<∞, the data are right-censored.

### 2.3. Restricted Mean Survival Times

#### 2.3.1. Problem Formulation and Notation

- Event Time: let $T_{i}\geq 0$ denote the true (possibly unobserved) event time for the $i^{th}$ subject.
- Observed Interval: due to intermittent assessment, we only observe a random interval $\left[L_{i},R_{i}\right]$ such that $P(T_{i}\in \left[L_{i},R_{i}\right])=1$, covering left-, right-, and interval-censoring (Section 2.2).
- RMST: for a pre-specified, clinically meaningful upper bound of the time window τ, the RMST μ(τ) is defined as the expected survival time restricted to [0,τ]:
  $$\mu \left(\tau \right)=E\left[\min\left(T,\tau \right)\right]=\int_{0}^{\tau }S\left(t\right)dt,$$
  where S(t)=P(T>t) is the survival function.

#### 2.3.2. Construction of the Response Variable via Pseudo-Values

To handle interval-censored data and obtain a response variable suitable for regression analysis, we generate RMST pseudo-observations for each individual through the following three-step procedure.

Step 1: Survival Function Estimation via EM-ICM.

Given interval-censored data ${\left\{\left(L_{i},R_{i}\right]\right\}}_{i=1}^{n}$, we obtain the nonparametric maximum likelihood estimator (NPMLE) of the survival function S(t) using the hybrid EM-ICM algorithm [20,21,22], which combines Expectation-Maximization (EM) and Iterative Convex Minorant (ICM) algorithms for optimizing non-increasing functions. Let ${\left\{s_{j}\right\}}_{j=0}^{m}$ be the ordered unique points from the set ${\left\{0,L_{i},R_{i}\right\}}_{i=1}^{n}$, and define:

$$\alpha_{ij}=I\left(s_{j}\in \left(L_{i},R_{i}\right]\right),\;\rho_{j}=S\left(s_{j-1}\right)-S\left(s_{j}\right).$$

The likelihood function is:

$$L_{s}\left(\rho \right)=\prod_{i=1}^{n}\left[S\left(L_{i}\right)-S\left(R_{i}\right)\right]=\prod_{i=1}^{n}\sum_{j=1}^{m}\alpha_{ij}\rho_{j}.$$

The product form arises because $S\left(L_{i}\right)-S\left(R_{i}\right)$ (the probability that $T_{i}\in \left[L_{i},R_{i}\right]$) equals the sum of $\rho_{j}$ for all intervals $\left(s_{j-1},s_{j}\right]$ contained within $\left[L_{i},R_{i}\right]$.

The EM-ICM algorithm estimates ρ (and thus S(t)) via iterative updates until convergence:

- E-step (Expectation): Since $T_{i}$ is unobserved (only $\left[L_{i},R_{i}\right]$ is known), we compute the expected number of events at each time point $s_{j}$, given the current estimate of ρ (denoted $\rho^{\left(c\right)}$). This expectation is quantified by:
  $$d_{j}\left(\rho^{\left(c\right)}\right)=\sum_{i=1}^{n}\frac{\alpha_{ij}\rho_{j}^{\left(c\right)}}{\sum_{l=1}^{m}\alpha_{il}\rho_{l}^{\left(c\right)}}.$$
- M-step (Iterative Convex Minorant, ICM): This step updates ρ to maximize the log-likelihood while ensuring the survival function S(t) remains valid. First, we convert $\rho_{j}$ to cumulative distribution function (CDF) estimates $\beta_{j}=\sum_{l=1}^{j}\rho_{l}$ (where $\beta_{j}=1-S\left(s_{j}\right)$, requiring non-decreasing $\beta_{j}$), then use the Pool Adjacent Violators Algorithm (PAVA) to solve the constrained optimization of maximizing a quadratic approximation of the log-likelihood under $\beta_{1}\leq \beta_{2}\leq \cdots \leq \beta_{m}$. Finally, we obtain the survival function estimator as $\hat{S}\left(t\right)=1-{\hat{\beta }}_{j}$ for $t\in [s_{j-1},s_{j})$. Detailed steps can be found in Section 3 of Sun [19].

Step 2: Population-level RMST Calculation

Using the estimated survival function $\hat{S}\left(t\right)$ (obtained from all *n* subjects), we compute the population-level RMST at each time point $\tau_{k}$ (the average survival duration of the entire cohort within $\left[0,\tau_{k}\right]$):

$$\hat{\mu }\left(\tau_{k}\right)=\int_{0}^{\tau_{k}}\hat{S}\left(t\right)\;dt.$$

Step 3: Individual-level RMST Generation

To derive individual-specific RMST estimates for each subject *i* and time point $\tau_{k}$, we employ the Jackknife method [9]:

(1) $${\tilde{y}}_{i}\left(\tau_{k}\right)=n\cdot \hat{\mu }\left(\tau_{k}\right)-\left(n-1\right)\cdot {\hat{\mu }}_{\left(-i\right)}\left(\tau_{k}\right),$$

where ${\hat{\mu }}_{\left(-i\right)}\left(\tau_{k}\right)$ is the ‘leave-one-out’ estimator computed after excluding the $i^{th}$ subject. The pseudo-values $\left\{{\tilde{y}}_{i}\left(\tau_{k}\right)\right\}$ form a fully-observed, continuous response suitable for standard regression modeling.

### 2.4. Network Architecture

#### 2.4.1. Build Connection Graph

This study employs GCN layers to model the relationships and neighborhood information among subjects. A connection graph is first constructed to serve as the input topology for the GCN. In this graph, each node represents a subject (totaling 744 nodes in our cohort), characterized by its gene expression profile as detailed in Section 3.3.1. An undirected edge with a uniform weight of 1 is established between a pair of subjects if one is among the $k_{n}$-nearest neighbors of the other, signifying sufficient gene expression similarity without further weighting. Subject similarity is quantified using the Euclidean distance, computed as $D_{ij}=\sqrt{\sum_{m=1}^{p}{\left(X_{im}-X_{jm}\right)}^{2}}$, where $X_{im}$ denotes the expression value of the $m^{th}$ gene for subject *i*. A smaller $D_{ij}$ indicates higher similarity in expression patterns. The graph construction relies on the K-nearest neighbors (KNN) algorithm, which connects each node to its $k_{n}$ closest neighbors, with the neighborhood parameter $k_{n}$ controlling the local connectivity density. The optimal neighborhood size $k_{n}$ was determined via grid search combined with cross-validation on the training data, selecting from the candidate set {5, 10, 15, 20} based on MSE. This process yields an adjacency matrix that encodes these connections, where an entry of 1 indicates the presence of an edge and 0 its absence.

#### 2.4.2. Graph Convolution Network with Decision Trees

This study designed a neural network architecture that integrates GCNs with stochastic neural decision trees [17] for survival prediction. The integration specifically addresses two needs in studies: (1) GCNs overcome limitations of conventional models in capturing complex interaction networks through graph-structured learning, while (2) the stochastic decision trees provide probabilistic branching at split nodes to enhance decision-making interpretability that pure deep learning models may lack. By jointly optimizing the global objective function, this framework preserves interpretable decision pathways while enhancing the predictive performance in survival analysis.

GCNs extend the convolution operator to the graph domain, demonstrating significant performance in various domains by effectively aggregating information among nodes. The essence of the GCN layer lies not in gathering information from the original nodes but in aggregating information from nodes linked by edges. The computation of the GCN layer is as follows:

$$H^{\left(\nu +1\right)}=f\left(H^{\left(\nu \right)},A\right)=\sigma \left(\hat{A}H^{\left(\nu \right)}W^{\left(\nu \right)}\right).$$

Here, ν indicates the layer index, and $W^{\left(\nu \right)}$ denotes the trainable parameter matrix. $\hat{A}$ is a symmetric normalization of the graph’s adjacency matrix, defined as ${\tilde{D}}^{-1/2}\tilde{A}{\tilde{D}}^{-1/2}$, where $\tilde{A}$ and $\tilde{D}$ represent the adjacency and degree matrices of the connection graph, respectively. The adjacency matrix $\tilde{A}$ is obtained by adding self-connections to the original adjacency matrix *A*, which is achieved through the operation $\tilde{A}=A+I$, where *I* denotes the identity matrix. The activation function σ(·) is the Rectified Linear Unit [23] (ReLU). $H^{\left(\nu \right)}$ represents the input matrix of the $v^{th}$ GCN layer, with $H^{\left(0\right)}=X$, where *X* denotes the gene expression matrix.

Using multiple layers of GCNs enables the aggregation of multi-order neighbor information. A two-layer GCN is employed, yielding the following outcomes:

$$H^{\left(2\right)}=f\left(f\left(X,A\right),A\right)=ReLU\left(\hat{A}ReLU\left(\hat{A}XW^{\left(0\right)}\right)W^{\left(1\right)}\right).$$

**Figure 1:**
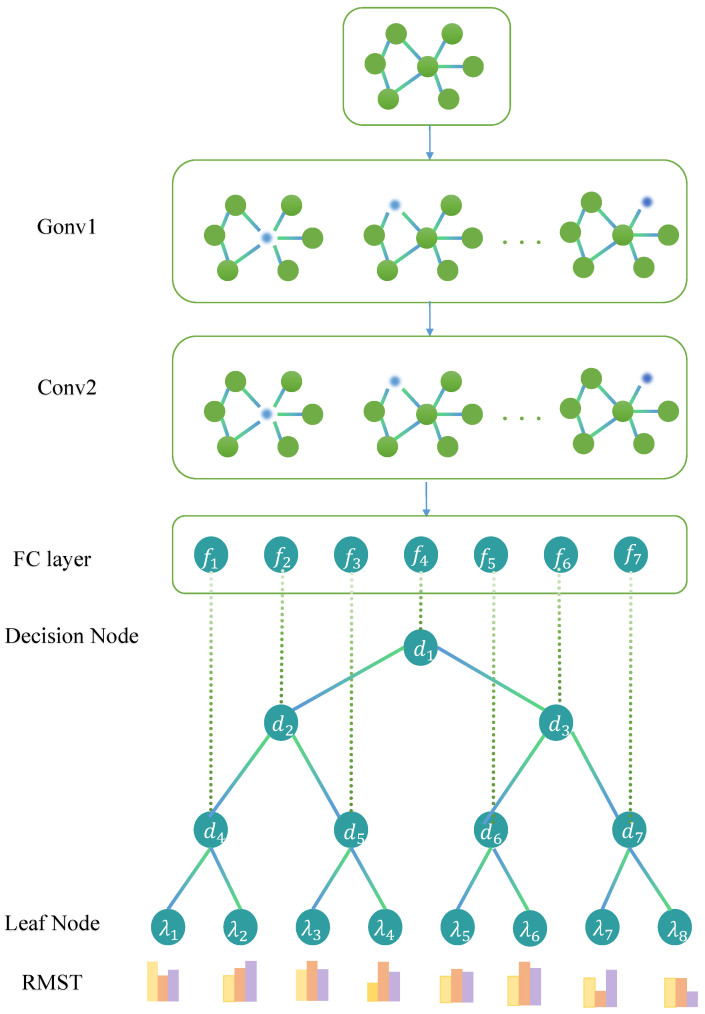
Illustration of the proposed GTsurvival architecture, comprising two GCN layers and a neural decision tree. FC layer: The fully connected (FC) layer provides the function $f_{e}\left(x;\theta \right)$. Decision Node: Each decision node e∈E corresponds to a split node within a tree, yielding the routing decision $d_{e}\left(x,\theta \right)=\sigma \left(f_{e}\left(x;\theta \right)\right)$ that dictates whether the sample *x* is assigned to the left or right subtree. Leaf Node: Circles at the bottom represent leaf nodes, offering RMST predictions at *K* time points.

Then a back propagation-compatible decision tree is incorporated into GCNs to guide representative learning in deeper layers. The structure of the GTsurvival network is depicted in Figure 1.

Decision trees consist of internal decision nodes and leaf nodes. Here, *E* represents the set of internal decision nodes, and *L* denotes the set of leaf nodes situated at the bottom of the tree. Each decision node e∈E is associated with a parameterized decision function $d_{e}\left(\cdot ;\Theta \right):X\to \left[0,1\right]$, guiding samples through the tree:

$$d_{e}\left(X_{i};\Theta \right)=d_{s}\left(f_{e}\left(X_{i};\Theta \right)\right),$$

where $d_{s}\left(x\right)={\left(1+e^{-x}\right)}^{-1}$ stands for the sigmoid function, and $f_{e}\left(\cdot ;\Theta \right)$ constitutes a real-valued mapping from the previous network layer. As a sample $X_{i}$ arrives at an internal decision node *e*, the value of $d_{e}\left(X_{i},\Theta \right)$ determines its subsequent routing, either to the left or right subtree.

To provide an explicit form for the routing function, the following binary relations are introduced based on the tree’s structure:

$$p_{l}\left(X_{i}|\Theta \right)=\prod_{e\in E}d_{e}{\left(X_{i};\Theta \right)}^{I_{l↙e}}{\bar{d}}_{e}{\left(X_{i};\Theta \right)}^{I_{e↘l}},$$

where the sample $X_{i}$ is assigned to either the left branch (denoted as l↙e) or the right branch (denoted as e↘l) of the subtree. Here, ${\bar{d}}_{e}\left(x;\Theta \right)=1-d_{e}\left(x;\Theta \right)$, with $I_{l↙e}$ representing the indicator function.

The routing function $p_{l}\left(X_{i}|\Theta \right)$ dictates the path for sample $X_{i}$ to reach leaf node *l*. Upon reaching the $l^{th}$ leaf node, the corresponding RMST prediction at the $k^{th}$ time point ($\tau_{k}$) is given by $\lambda_{lk}$, and $\Lambda =\sum_{l=1}^{L}\sum_{k=1}^{K}\lambda_{lk}$. Predictions from all *L* leaf nodes are then aggregated, yielding the final prediction for sample $X_{i}$:

(2) $$g\left(X_{i},\tau_{k};\Theta ,\Lambda \right)=\sum_{l\in L}\lambda_{lk}p_{l}\left(X_{i}|\Theta \right).$$

By integrating the RMST contributions from all leaf nodes as in Equation (2), the model predicts the future RMST for subject *i* at the $k^{th}$ observation time point.

#### 2.4.3. Loss Function

- Network output: for the $i^{th}$ subject at time $\tau_{k}$, the model output $g(X_{i},\tau_{k};\Theta ,\Lambda )$ is the predicted RMST, computed by aggregating contributions from all leaf nodes of the neural decision tree (Equation (2) in the manuscript).
- Training objective: The core goal of the network is to minimize the difference between the predicted RMST $g(X_{i},\tau_{k};\Theta ,\Lambda )$ and the RMST pseudo-observations ${\tilde{y}}_{i}\left(\tau_{k}\right)$ (constructed via the Jackknife method as detailed in Section 2.3). For the $i^{th}$ subject, the loss (capturing prediction error across *K* time points) is:
  $${\mathrm{MSE}}_{i}=\sum_{k=1}^{K}{\left({\hat{y}}_{ik}-g\left(X_{i},\tau_{k};\Theta ,\Lambda \right)\right)}^{2}.$$
  The overall loss function for model training is the average mean squared error (MSE) across all subjects and time points:
  (3) $$L\left(\Theta ,\Lambda \right)=\frac{1}{nK}\sum_{i=1}^{n}{\mathrm{MSE}}_{i}=\frac{1}{nK}\sum_{i=1}^{n}\sum_{k=1}^{K}{\left({\hat{y}}_{ik}-g\left(X_{i},\tau_{k};\Theta ,\Lambda \right)\right)}^{2}.$$

#### 2.4.4. Gradient-Based Learning for Decision Tree Parameters

The key innovation of the GTsruvival model is its end-to-end joint optimization of the GCN and the neural decision tree. This integration is achieved by computing and backpropagating gradients through all trainable parameters of the decision tree module. This subsection details the learning mechanism for the decision tree parameters, the decision node parameters Θ and the leaf node parameters Λ. We derive the gradients that connect the global loss function to parameter updates, ensuring convergence within a unified optimization framework while preserving the interpretable structure of the decision tree. The gradient calculations rely on the chain rule to trace error signals from the loss function back to each parameter (see Section S3 of the Supplementary Document for the complete derivation). We present the final gradient expressions below and discuss their roles in the training dynamics.

(1) Gradient for Decision Nodesr

The gradient for a decision node parameter Θ is a function of three components: the prediction error, the node’s classification confidence, and the performance difference between its subtrees. The gradient formula is:

$$\frac{\partial L(\Theta ,\Lambda )}{\partial f_{e}\left(X_{i};\Theta \right)}=\frac{2}{nK}\cdot d_{e}\left(X_{i};\Theta \right)\cdot {\bar{d}}_{e}\left(X_{i};\Theta \right)\cdot \sum_{k=1}^{K}\left(r_{ik}\cdot \left(A_{e_{r}}^{\left(k\right)}-A_{e_{l}}^{\left(k\right)}\right)\right)$$

where $r_{ik}=g_{i}\left(X_{i},\tau_{k};\Theta ,\Lambda \right)-{\hat{y}}_{ik}$ is the prediction residual for the $i^{th}$ sample at the $k^{th}$ time point. The term $d_{e}\cdot {\bar{d}}_{e}$ (with ${\bar{d}}_{e}=1-d_{e}$) quantifies the node’s decision uncertainty. $A_{e_{r}}^{\left(k\right)}$ and $A_{e_{l}}^{\left(k\right)}$ denote the final output of the right and left subtrees of node *e* at time $\tau_{k}$, respectively. Their difference $\left(A_{e_{r}}^{\left(k\right)}-A_{e_{l}}^{\left(k\right)}\right)$ indicates which subtree currently offers a better prediction. This gradient formula can be understood as comprising three instructional signals for the decision node. First, the term $d_{e}\cdot {\bar{d}}_{e}$ acts as an “attention” signal, which peaks when the node’s decision entropy is maximized ($d_{e}\approx 0.5$), directing learning effort toward the most uncertain splits. Second, the residual $r_{ik}$ serves as a “correction” signal, scaling updates proportionally to the magnitude of the prediction error. Third, the difference $\left(A_{e_{r}}^{\left(k\right)}-A_{e_{l}}^{\left(k\right)}\right)$ functions as a “guidance” signal, steering samples toward the better-performing subtree. By prioritizing high-entropy nodes while incorporating precise error correction and clear routing directions, this mechanism leads to efficient and targeted training.

(2) Gradient of Leaf Node Parameters

The gradient for leaf node parameter $\lambda_{lk}$ is:

$$\frac{\partial L(\Theta ,\Lambda )}{\partial \lambda_{lk}}=\frac{2}{nK}\cdot r_{ik}\cdot p_{l}\left(X_{i}|\Theta \right),$$

where $p_{l}\left(X_{i}|\Theta \right)$ is the routing probability of sample $X_{i}$ to leaf node *l* (governed by network parameters Θ), and $r_{ik}={\hat{y}}_{ik}-g\left(X_{i},\tau_{k};\Theta ,\Lambda \right)$ denotes the model’s prediction residual for sample $X_{i}$ at $\tau_{k}$. This gradient implements a probability-weighted update rule for leaf nodes: the update magnitude of $\lambda_{lk}$ is scaled by $p_{l}\left(X_{i}|\Theta \right)$. Leaves frequently visited by samples (with large $p_{l}\left(X_{i}|\Theta \right)$ values) get consistent updates. This lets them optimize predictions for common data. In contrast, rarely accessed leaves receive minimal adjustments, which prevents disruptive updates from sparse samples or outliers. The update magnitude is also modulated by the prediction residual $r_{ik}$. Meaningful parameter changes only occur when both prediction error and routing probability are high. This adaptive scaling focuses updates on the most relevant leaf-sample pairs, stabilizing model training and accelerating the convergence of the neural decision tree.

#### 2.4.5. Implementation Details

For evaluation, we apply fivefold cross-validation for the selection of key hyperparameters: we randomly split the data into a training set (80%) and a test set (20%), with 10% of the training set allocated as the validation set. The GTsurvival model consists of two GCN layers and a neural decision tree component. The model is trained by back-propagation with the Adam optimizer of a learning rate of 0.001 for 1000 epochs. The dropout probability of 0.1 and the weight decay of 1 are applied. Early stopping is performed based on the validation loss to avoid overfitting. Model weights are initialized using TensorFlow’s default scheme, and the loss function is mean squared error in Equation (3), which directly minimizes the difference between the RMST pseudo-observations and the network predictions.

The optimization of hyperparameters is performed individually for each fold by a grid search, and the configuration is selected such that the corresponding model achieves the best performance on the validation set. The search spaces for the hyperparameters are as follows: (1) For graph construction: the number of neighbors $k_{n}$ in the KNN algorithm is selected from the candidate set {5, 10, 15, 20}; (2) For GCN architecture: the dimensions of the two hidden layers are tuned within the range {[64, 32], [128, 64], [256, 128]}, with ReLU activation function applied after each layer; (3) For neural decision tree component: the depth of the tree is optimized from {2, 3, 4, 5}, where each decision node *e* is equipped with a learnable routing function $d_{e}\left(X_{i};\Theta \right)=d_{s}\left(f_{e}\left(X_{i};\Theta \right)\right)$ ($d_{s}$ denotes the sigmoid function, and $f_{e}$ is a fully connected layer that maps the GCN output to the decision tree), enabling soft decision-making where samples are assigned to left or right subtrees based on the value of $d_{e}$.

## 3. Results

### 3.1. Simulation and Experimental Design

Simulations were performed to evaluate the performance of the GTsurvival algorithm. In this study, GTsurvival was compared with several benchmark methods including: (i) a two-layer GCN without neural decision tree, (ii) Convolutional Neural Network (CNN) with two layers of convolution and pooling, and (iii) Deep Neural Network (DNN), to demonstrate its effectiveness. To ensure a fair comparison, all methods were optimized via grid search. Hyperparameter tuning was conducted within the fivefold cross-validation framework detailed in Section 2.4.5, adhering to the predefined search spaces, and optimal tuning parameters for each model were systematically selected based on the MSE metric. A fixed random seed was used to guarantee identical training/validation/test splits across all compared methods. Evaluation results were summarized over 100 replications to provide a robust performance assessment, and detailed descriptions of the three simulation settings are provided below.

Each simulated dataset was generated from a specified multivariate normal distribution MVN(0,Σ) with mean zero and a covariance matrix Σ where the (l,m) element was equal to $0.5^{\left|l-m\right|}$. The survival function was assumed to be $S\left(t|X\right)=\exp\left(-\eta (X)t\right)$, where η(X) in the three experiments is as follows:

Experiment 1:  $\eta_{1}\left(X\right)=|X_{1}|+{\left(X_{2}-0.5\right)}^{2}+\left|X_{3}-X_{4}\right|$,
Experiment 2:  $\eta_{2}\left(X\right)=\sum_{p=1}^{P}\gamma_{p}X_{p}$,
Experiment 3:  $\eta_{3}\left(X\right)=\sum_{p=1}^{P}\gamma_{p}X_{p}+X_{3}^{2}+X_{4}^{2}$,

where $\gamma_{p}$ follows $\mathrm{MVN}\left(0.2,0.01\Sigma \right)$. The failure time *T* was derived via the inverse probability sampling approach based on the respective survival functions from Experiments 1 to 3. For simulating censored data, 10 visits (K=10) were constructed, where $V_{1}$ follows a uniform distribution over the interval (0,2) and $V_{K}$ is defined as $V_{k}=V_{k-1}+U\left(0,1\right)$. Observations satisfying $T<V_{1}$ were treated as left-censored, with L=0 and $R=V_{1}$; those meeting $T>V_{K}$ were right-censored, with $L=V_{k}$ and R=∞; and observations falling into the range $V_{k-1}<T<V_{k}$ were classified as strictly interval-censored, where $L=V_{k-1},\;R=V_{k}$.

The proposed approach GTsurvival was evaluated by varying the sample size n=200/400 and features counts p=50/100/500. MSE and mean absolute error (MAE) metrics were employed to assess the performance of various methods, where

$$\begin{array}{cc} \; & \mathrm{MSE}=\frac{1}{n}\sum_{i=1}^{n}\sum_{k=1}^{K}{\left({\hat{y}}_{ik}-g\left(X_{i},\tau_{k};\Theta ,\Lambda \right)\right)}^{2},\\ \; & \mathrm{MAE}=\frac{1}{n}\sum_{i=1}^{n}\sum_{k=1}^{K}\left|{\hat{y}}_{ik}-g\left(X_{i},\tau_{k};\Theta ,\Lambda \right)\right|. \end{array}$$

Here ${\hat{y}}_{ik}$ denotes the observed RMST at the $k^{th}$ observed time point for the $i^{th}$ sample, and $g(X_{i},\tau_{k};\Theta ,\Lambda )$ denotes the corresponding predicted value from GTsurvival.

### 3.2. Simulation Results

Throughout the simulation process, *K* observation points were set for estimating RMST. For $K\in \left\{1,3,5\right\}$, the specific observation times $\left\{\tau_{1},\tau_{2},...,\tau_{K}\right\}$ were selected as {1}, {1,3,5}, and {1,3,5,7,9} years, respectively. Then the impact of various sample sizes n=200/400 and feature counts p=50/100/500 was explored on the predictive performance of the model. Model performance was evaluated across three groups of experiments, comparing GTsurvival against CNN, DNN, and GCN based on MSE and MAE metrics. Generally, a model with lower MSE and MAE values is considered more favorable.

In Experiment 1, Table 1 presented the MSE and MAE results of several algorithms. Supplementary Tables S1 and S2 corresponded to the results of Experiments 2 and 3. Figure 2 and Figure 3 illustrated box plots for Experiment 1 at K=3 to offer a clearer visualization of the data whilst box plots for the remaining simulation experiments were displayed in Supplementary Figures S1–S16.

Firstly, the different methods in Experiment 1 were compared, and the numerical results were listed in Table 1. As anticipated, GTsurvival significantly outperformed CNN, DNN and GCN, as evidenced by its lowest MSE and MAE values. Specifically, GTsurvival was superior to GCN, suggesting that combining a neural decision tree with GCN is more favorable than using GCN alone for survival analysis.

Secondly, as presented in Supplementary Table S1 for Experiment 2, GTsurvival exhibited superior performance on both metrics compared to other algorithms, validating its robust predictive capabilities. Despite marginally underperforming compared to GTsurvival, GCN outperformed CNN and DNN on the majority of simulated datasets, highlighting the significant role of GCN’s graph structure in the network’s functionality.

Thirdly, GTsurvival exhibited strong generality in Experiment 3, even with varying samples and feature counts. Meanwhile, GTsurvival and GCN exhibited comparable MSE metrics at K=3. Except for (n,p)=(400,500), where GCN (MAE = 0.646) performed slightly better than GTsurvival (MAE = 0.651), GTsurvival outperformed the other models in all other settings. Therefore, these results collectively indicated that GTsurvival provided a reliable approach to analyzing and predicting survival data.

### 3.3. Application

#### 3.3.1. Data Preprocessing and Genetic Feature Selection

We applied the GTsurvival method outlined in Section 2 to analyze a genomic dataset from ADNI. First, raw gene expression values extracted directly from Affymetrix HG U219 Array CEL files were preprocessed using the Robust Multi-chip Average (RMA) algorithm, which includes background correction, quantile normalization, and probe set summarization to ensure cross-sample comparability. Following this, we discarded genes with missing or zero expression across all subjects to obtain the initial expression matrix. Subsequently, to select genetic features associated with disease progression prior to graph construction and model training, we adopted the generalized score test proposed by Sun and Ding [10]. Setting *p*-value thresholds at 0.02, 0.005, and 0.002 resulted in 592, 175, and 80 genetic features, respectively. These selected features were used for subsequent graph construction and model training.

When applying the generalized score test for feature selection, our objective was to identify genetic variants that maximize the reduction of uncertainty in predicting disease progression. This selection process aligns with entropy-constrained information extraction in information theory: by testing the statistical association between genetic variants and disease progression, the test selects features with the highest information contribution to survival prediction and eliminates redundant or irrelevant variations. These selected features (i.e., genes) convey the most survival outcome-relevant information, enabling subsequent graph neural networks to learn efficiently on a more information-dense feature subset.

#### 3.3.2. Construction of Gene Interaction Network

**Figure 4:**
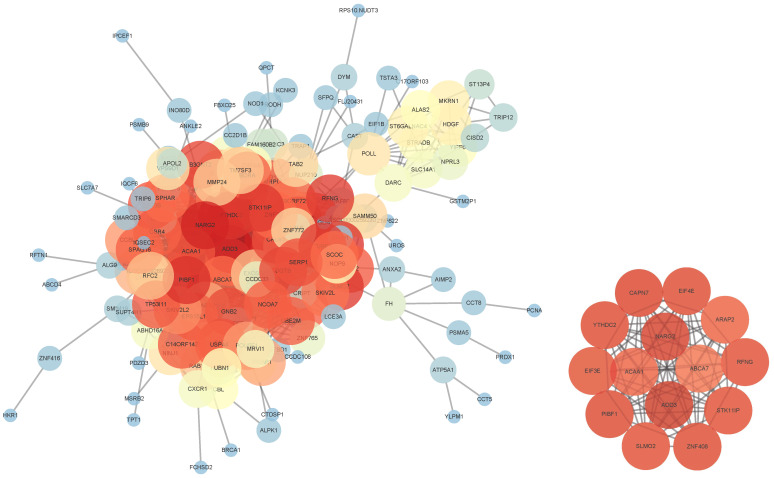
The gene interaction network diagram with a significance threshold of the *p*-value < 0.02. Pearson correlation coefficients are utilized in the main figure to define the network’s edges, providing a clear representation of gene associations. The subgraph in the bottom right corner showcases the sub-networks derived from the genes ADD3, ACAA1, NARG2, and ABCA7. Nodes are color-coded based on their degree of connectivity: red points indicate nodes with high degrees, representing strong relationships with other genes, while blue points indicate nodes with low degrees, representing weaker relationships.

**Figure 5:**
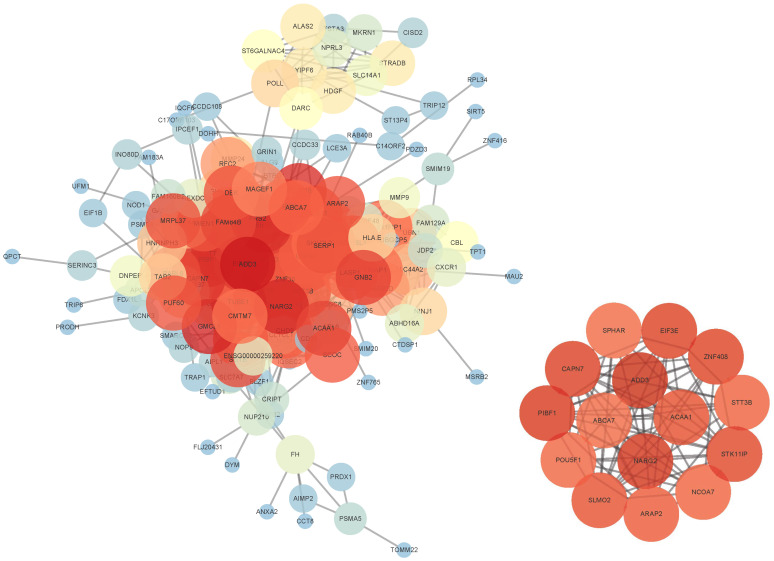
This gene interaction network diagram with a significance threshold of the *p*-value < 0.02. Spearman correlation coefficients were used to establish the network’s edges, ensuring a robust representation of gene associations. A detailed subgraph, positioned in the bottom-right corner, highlights sub-networks originating from the genes ADD3, ACAA1, NARG2, and ABCA7. Nodes are color-coded based on their connectivity: red points indicate nodes with high degrees, signifying strong associations with other genes, while blue points represent nodes with low degrees, indicating fewer connections.

A group of representative genes with *p*-value < 0.02 were extracted, and the similarity of gene expression patterns across all samples was measured using Pearson and Spearman correlation coefficients. Cytoscape (version 3.10.0) was utilized to construct the gene interaction network, which is open source and freely available from https://cytoscape.org/ (accessed on 5 March 2025), under the GNU LGPL (Lesser General Public License). In the network, edges between genetic nodes were connected when the correlation coefficient between gene pairs exceeded 0.6. Notably, this study specifically explored the genes ABCA7, ADD3, NARG2, and ACAA1, as identified in Figure 4 and Figure 5. Evidence from human-based studies [24] has confirmed ABCA7 as one of the most prominent genes linked to AD risk, harboring both common and rare risk variants with a substantial impact on AD risk. Notably, ADD3, alternatively known as Adducin 3, is a protein-coding gene involved in disorders including cerebral palsy, spastic quadriplegic 3, and spastic quadriplegic cerebral palsy. Liang et al. [25] conducted a weighted gene co-expression network analysis and indicated that it could be a potential therapeutic target for AD. Furthermore, genetic analyses [26] of cortical grey matter thickness and fractional anisotropy showed a notable correlation between the NARG2 gene and gray matter. In addition, a recent study [27] discovered a novel missense variant in ACAA1, linked to early-onset AD, impaired lysosomal function, and the worsening of amyloid-β pathology and cognitive decline. These novel findings may aid in enhancing the comprehension of AD pathogenesis.

#### 3.3.3. Model Training and Performance Comparison

We developed a predictive model GTsurvival using representative genetic features. The ADNI dataset was evaluated under the same fivefold cross-validation described in Section 2.4.5. All methods were applied to the same data splits. Hyperparameter optimization used the predefined search spaces from Section 2.4.5, ensuring a consistent and fair comparison across all models. In addition to the methods in Section 3.1, we further include FastPseudo [8] for comparison. This model accelerates convergence via asymptotic formulas for computing pseudo-observations of the survival function and RMST under right-censored and interval-censored data.

Table 2 presented the results of GTsurvival across various *p*-value ranges. For *p*-values less than 0.02, GTsurvival outperformed other models, demonstrating the lowest MSE and MAE metrics. In contrast, CNN performed poorly in both MSE and MAE metrics with *p*-value less than 0.005. Overall, GTsurvival outperformed other models in the majority of scenarios. For *p*-values less than 0.002, GTsurvival achieved the lowest MAE value of 0.577, which was 35.1% lower than that of DNN. To verify whether the improvements of the proposed method over other models are statistically significant, we also conducted Wilcoxon signed-rank tests. The detailed results are presented in Supplementary Table S3. Notably, all corresponding *p*-values from the Wilcoxon tests are less than 0.01, confirming that the performance advantages of our proposed GTsurvival model over baseline methods are statistically significant.

#### 3.3.4. Interpretation of Results via LIME

**Figure 6:**
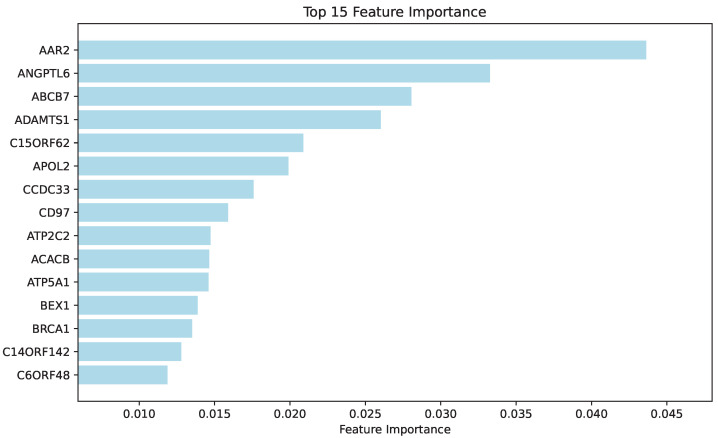
Feature importance plot based on LIME method.

To interpret GTsurvival’s predictions, we applied Local Interpretable Model-agnostic Explanations [16] (LIME) to evaluate predictor importance. This approach constructs simplified local models that approximate the behavior of complex neural networks. The top 15 important predictors are visualized in Figure 6. Our analysis identified several notable genes, including ADAMTS1, ACACB, and BRCA1. Existing evidence supports their roles in neurodegenerative processes. Specifically, Gurses et al. [28] demonstrated that ADAMTS genes play a significant role in neuroplasticity regulation and are implicated in nervous system pathologies including AD. These findings suggest therapeutic potential for ADAMTS family members in addressing central nervous system disorders, ischemic injuries, and neurodegenerative conditions. Furthermore, Liu et al. [29] identified ACACB as a hub gene through protein-protein interaction network analysis, suggesting its potential diagnostic value as an AD biomarker. The BRCA1 gene, known for its involvement in DNA damage response (DDR) mechanisms, has been linked to the pathogenesis of AD. Nakamura et al. [30] proposed that DDR dysfunction resulting from cytoplasmic sequestration of BRCA1 is involved in the pathogenesis of tauopathies. These findings may provide insights into the mechanisms of AD.

#### 3.3.5. External Validation on TCGA-LGG Dataset

To further validate the predictive performance, we also analyzed the brain lower-grade glioma (LGG) dataset from The Cancer Genome Atlas (TCGA). This dataset contains 531 samples and 20,530 genetic features, with patient death as the event of interest. To reduce the influence of noise-sensitive features, we first filtered out genetic features with very low variation across samples (as measured by variance). The remaining features were then ranked by information gain relative to the outcome variable in descending order, and the top 600 genes were selected for further analysis. As shown in Supplementary Table S4, GTsurvival demonstrates better prediction performance compared to GCN, FastPseudo, and other methods.

#### 3.3.6. Ablation Study

To verify the contribution of the dual fusion strategy in GTsurvival to survival prediction, we conducted ablation experiments on the ADNI and TCGA-LGG datasets by modifying different components of the model configuration. We adopted the following configurations to evaluate the effectiveness of each component:

- GCNet: Only graph convolutional network (GCN) layers, without the neural decision tree.
- NDF: Two fully connected (FC) layers combined with the neural decision tree (no GCN layers).
- FastPseudo: Utilizes the RMST estimation method proposed by Bouaziz [8], instead of the method described in Section 2.3.

As shown in Supplementary Table S5, GTsurvival integrates GCN and neural decision tree via the dual fusion strategy and achieves the best performance on both datasets, with smaller MAE and MSE indicating superior predictive ability.

## 4. Discussion and Concluding Remarks

Chronic progressive diseases such as AD are multifactorial disorders driven by intricate interactions between genetic variants and environmental factors. Traditional linear models are often limited in their ability to capture non-linear interactions and high-dimensional genomic complexity, which can lead to a loss of critical biological signals relevant to disease progression. Our proposed model, GTsurvival, which integrates GCNs with the neural decision tree, addresses these limitations by leveraging graph-structured learning to model genetic interactions and using pseudo-observations based on RMST to handle censored data effectively. The improved performance of GTsurvival over conventional methods, demonstrated in both simulated data and the ADNI cohort, supports its potential to advance the early prediction of diseases.

A key advantage of GTsurvival is its ability to identify genetic variants strongly associated with AD progression through generalized score testing and feature importance analysis (Figure 6). The most significant genes identified include ABCA7, ADD3, NARG2, ACAA1, ADAMTS1, ACACB and BRCA1, each of which has a documented role in AD-related biological processes, as supported by prior literature and reinforced by our network analysis (Figure 4 and Figure 5). For example, ABCA7 is a well-recognized AD risk gene [24]. A recently identified missense variant in ACAA1 was shown to impair lysosomal function, thereby aggravating amyloid-β pathology and accelerating cognitive decline in early-onset AD [27]. Other genes also contribute to key dysfunctional processes in AD: ADAMTS1 is implicated in neuroplasticity [28], ACACB has been proposed as a potential biomarker [29], and BRCA1 participates in DNA damage repair mechanisms relevant to disease pathogenesis [30]. Together, these findings confirm that GTsurvival effectively captures biologically meaningful signals relevant to disease progression.

Noteworthily, survival prediction remains a challenging field requiring further optimization despite the significant predictive capability of GTsurvival. First, the model was developed and validated on the genetic dataset; integrating additional multimodal data (e.g., DNA methylation or neuroimaging features) could improve its accuracy and generalizability. Second, the predominantly White cohort limits the extrapolation of findings [31], as AD mechanisms may vary across ethnicities, and future work should include more diverse populations. Third, the graph construction relies on KNN with uniform edge weights, which may overlook variations in interaction strength between genetic features and limit the GCN’s ability to capture key biological relationships. Finally, expanding other evaluation metrics would provide a more comprehensive assessment of model performance. Addressing these aspects will be important for further refining and validating the model.

## Figures and Tables

**Figure 2:**
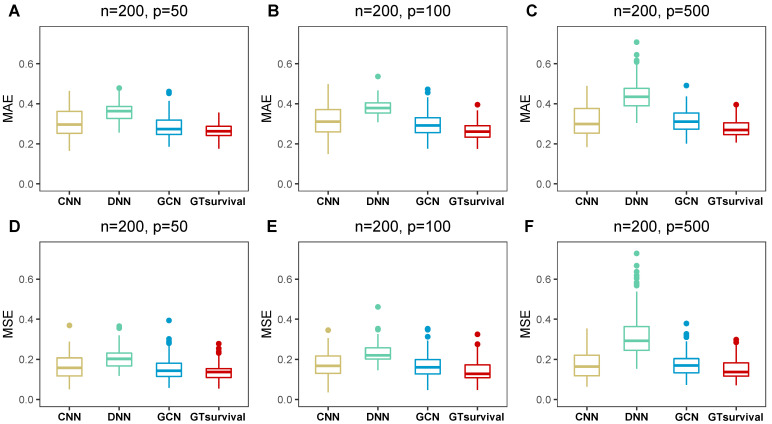
Box plots of MAE and MSE metrics in Experiment 1. Subfigures (**A**–**F**) correspond to the results of different feature count (*p*) settings (50, 100, 500) with a fixed sample size n=200 in Experiment 1, where (**A**–**C**) present the MAE metric results and (**D**–**F**) present the MSE metric results across the three time points ($\tau_{1}=1$, $\tau_{2}=3$, $\tau_{3}=5$ years).

**Figure 3:**
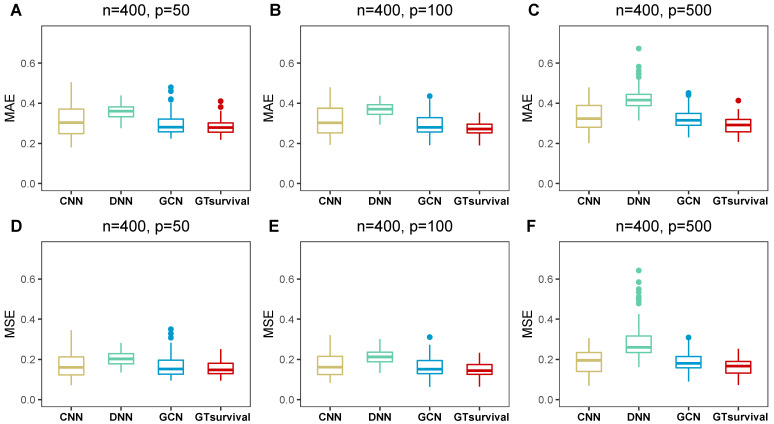
Box plots of MAE and MSE metrics in Experiment 1. Subfigures (**A**–**F**) correspond to the results of different feature count (*p*) settings (50, 100, 500) with a fixed sample size n=400 in Experiment 1, where (**A**–**C**) present the MAE metric results and (**D**–**F**) present the MSE metric results across the three time points ($\tau_{1}=1$, $\tau_{2}=3$, $\tau_{3}=5$ years).

**Table 1:** Simulation experiments on GTsurvival and the other three methods in Experiment 1. The sample sizes were set as n=200/400, and feature counts were set as p=50/100/500. The RMST was estimated at *K* time points, where K∈{1,3,5}. The specific observation times $\tau_{1},\tau_{2},\ldots ,\tau_{K}$ were selected as follows: {1}, {1,3,5}, and {1,3,5,7,9} years. The employed evaluation metrics are MSE and MAE, where smaller values indicate better prediction performance. The top performance is highlighted in bold, and the second-top result is underlined.

		MAE (SD)			MSE (SD)		
	Method	p=50	p=100	p=500	p=50	p=100	p=500
K=1							
n=200	DNN	0.305 (0.122)	0.333 (0.121)	0.415 (0.125)	0.144 (0.072)	0.168 (0.070)	0.297 (0.073)
	CNN	0.245 (0.050)	0.259 (0.051)	0.279 (0.055)	0.091 (0.039)	0.098 (0.040)	0.111 (0.066)
	GCN	0.200 (0.026)	0.214 (0.029)	0.247 (0.087)	0.063 (0.012)	0.071 (0.016)	0.091 (0.052)
	GTsurvival	**0.149 (0.022)**	**0.156 (0.019)**	**0.167 (0.029)**	**0.034 (0.009)**	**0.038 (0.008)**	**0.043 (0.013)**
n=400	DNN	0.273 (0.123)	0.305 (0.124)	0.378 (0.127)	0.114 (0.062)	0.142 (0.073)	0.232 (0.075)
	CNN	0.234 (0.048)	0.252 (0.045)	0.278 (0.055)	0.085 (0.034)	0.095 (0.036)	0.111 (0.062)
	GCN	0.185 (0.017)	0.198 (0.024)	0.230 (0.021)	0.055 (0.008)	0.062 (0.012)	0.080 (0.014)
	GTsurvival	**0.141 (0.013)**	**0.150 (0.021)**	**0.161 (0.017)**	**0.030 (0.005)**	**0.034 (0.008)**	**0.040 (0.008)**
K=3							
n=200	DNN	0.359 (0.077)	0.380 (0.079)	0.451 (0.082)	0.205 (0.059)	0.232 (0.062)	0.351 (0.069)
	CNN	0.304 (0.042)	0.313 (0.035)	0.320 (0.067)	0.162 (0.043)	0.172 (0.042)	0.174 (0.076)
	GCN	0.283 (0.050)	0.297 (0.045)	0.316 (0.050)	0.153 (0.046)	0.169 (0.049)	0.177 (0.045)
	GTsurvival	**0.264 (0.039)**	**0.266 (0.042)**	**0.280 (0.046)**	**0.138 (0.036)**	**0.144 (0.045)**	**0.149 (0.045)**
n=400	DNN	0.359 (0.076)	0.370 (0.075)	0.425 (0.076)	0.204 (0.059)	0.214 (0.059)	0.297 (0.059)
	CNN	0.312 (0.035)	0.316 (0.032)	0.334 (0.044)	0.171 (0.034)	0.172 (0.033)	0.189 (0.058)
	GCN	0.296 (0.042)	0.292 (0.047)	0.323 (0.043)	0.167 (0.044)	0.160 (0.042)	0.188 (0.042)
	GTsurvival	**0.283 (0.034)**	**0.275 (0.033)**	**0.292 (0.038)**	**0.156 (0.037)**	**0.148 (0.034)**	**0.164 (0.037)**
K=5							
n=200	DNN	0.367 (0.059)	0.381 (0.054)	0.436 (0.059)	0.222 (0.052)	0.239 (0.047)	0.324 (0.055)
	CNN	0.314 (0.037)	0.322 (0.027)	0.323 (0.046)	0.183 (0.035)	0.191 (0.034)	0.185 (0.058)
	GCN	0.277 (0.046)	0.292 (0.046)	0.307 (0.042)	0.151 (0.039)	0.165 (0.046)	0.169 (0.039)
	GTsurvival	**0.258 (0.040)**	**0.265 (0.042)**	**0.270 (0.037)**	**0.136 (0.040)**	**0.145 (0.045)**	**0.142 (0.037)**
n=400	DNN	0.382 (0.053)	0.382 (0.057)	0.422 (0.041)	0.240 (0.052)	0.238 (0.054)	0.293 (0.052)
	CNN	0.335 (0.032)	0.336 (0.028)	0.348 (0.032)	0.205 (0.039)	0.204 (0.030)	0.217 (0.038)
	GCN	0.299 (0.042)	0.297 (0.040)	0.320 (0.040,)	0.174 (0.038)	0.171 (0.037)	0.190 (0.041)
	GTsurvival	**0.284 (0.030)**	**0.279 (0.034)**	**0.291 (0.033)**	**0.162 (0.035)**	**0.158 (0.035)**	**0.169 (0.035)**

**Table 2:** Application results of GTsurvival and three comparative methods across different *p*-value ranges. The restricted mean survival time (RMST) was estimated at *K* time points (K∈{1,3,5}), with observation times $\tau_{1},\tau_{2},\ldots ,\tau_{K}$ set as {1}, {1,3,5}, and {1,3,5,7,9} years, respectively. Evaluation metrics include mean squared error (MSE) and mean absolute error (MAE), where smaller values indicate better predictive performance. The best performance is highlighted in bold, and the second-best result is underlined.

	MAE			MSE		
	K=1	K=3	K=5	K=1	K=3	K=5
*p*-value < 0.02						
FastPseudo	1.924 (0.243)	1.509 (0.519)	1.718 (0.481)	5.948 (1.467)	4.693 (3.306)	5.504 (3.137)
DNN	1.352 (0.885)	1.547 (0.479)	1.187 (0.376)	3.105 (1.953)	5.760 (3.518)	4.047 (2.608)
CNN	1.607 (0.936)	1.800 (0.470)	1.775 (0.304)	3.730 (2.043)	7.787 (3.536)	6.820 (2.384)
GCN	1.315 (0.837)	1.435 (0.430)	1.330 (0.345)	2.986 (1.846)	4.941 (3.062)	4.896 (2.413)
GTsurvival	**0.587 (0.094)**	**1.030 (0.142)**	**1.033 (0.159)**	**0.533 (0.265)**	**2.682 (1.362)**	**2.416 (0.942)**
*p*-value < 0.005						
FastPseudo	2.396 (0.833)	1.523 (0.484)	1.707 (0.467)	8.747 (7.876)	4.746 (3.311)	5.343 (2.938)
DNN	1.534 (0.895)	1.366 (0.483)	1.211 (0.321)	3.673 (1.650)	4.558 (2.891)	4.465 (2.488)
CNN	1.607 (0.770)	1.800 (0.470)	1.687 (0.306)	3.732 (1.854)	7.980 (3.538)	6.146 (2.398)
GCN	0.592 (0.180)	1.777 (0.407)	1.433 (0.385)	0.546 (0.207)	7.927 (3.310)	5.093 (2.665)
GTsurvival	**0.581 (0.089)**	**1.013 (0.131)**	**1.025 (0.150)**	**0.532 (0.263)**	**2.930 (1.324)**	**2.406 (0.891)**
*p*-value < 0.002						
FastPseudo	2.000 (0.453)	1.362 (0.408)	1.570 (0.400)	6.010 (3.968)	3.965 (2.615)	4.803 (2.677)
DNN	0.889 (0.547)	1.499 (0.486)	1.192 (0.295)	1.446 (1.910)	6.172 (2.602)	3.390 (2.045)
CNN	1.424 (0.775)	1.748 (0.340)	1.799 (0.297)	3.122 (1.610)	7.760 (3.554)	6.624 (2.321)
GCN	1.117 (0.619)	1.484 (0.467)	1.413 (0.302)	2.343 (1.866)	6.419 (3.614)	4.878 (2.478)
GTsurvival	**0.577 (0.088)**	**1.063 (0.133)**	**0.994 (0.122)**	**0.515 (0.237)**	**2.749 (1.388)**	**2.252 (0.898)**

## Data Availability

The dataset in this paper is from https://adni.loni.usc.edu/ (accessed on 5 March 2025). One can registered for a download account and apply for the data.

## Supplementary Materials

The following supporting information can be downloaded at: https://www.mdpi.com/article/10.3390/e28010028/s1. The following are available online. We evaluated the performance of GTsurvival and other methods in Experiments 1–3. Supplementary Figures S1–S4 are designed for Experiment 1. We observed the performance of GTsurvival in a variety of sample sizes n=200/400 and feature counts p=50/100/500. The results of Supplementary Figures S1–S4 indicated that GTsurvival can apply to datasets with non-linear relationship. Supplementary Figures S5–S10 are used to observe the generalization capability of GTsurvival in Experiment 2. GTsurvival performed best even under the different *K* groups. As for Experiment 3, GTsurvival obtained lower MSE and MAE than other methods in Supplementary Figures S11–S16. Supplementary Tables S1 and S2 presented the MSE and MAE results of several algorithms used in Experiments 2 and 3. The results indicated the advantage of GTsurvival in predicting survival in complex censored data. Supplementary Table S3. Statistical test results of GTsurvival vs. Second-Top baseline methods. Supplementary Table S4. Comparison of predictive performance among different methods on the LGG dataset (smaller values of MAE and MSE indicate better performance). Supplementary Table S5. Results of ablation experiments on ADNI and TCGA-LGG datasets. Section S3. Detailed process of gradient derivation.
