# Supplementary material for: GTsurvival: A Hybrid GCN-Neural Decision Tree Model for Restricted Mean Survival Time Prediction with Complex Censored Data
Source: Entropy (Basel). 2025 Dec 25;28(1):28. doi: 10.3390/e28010028 (PMC12840287; doi:10.3390/e28010028)
Supplement: Supplementary file 1 [file entropy-28-00028-s001.zip › entropy-4028882-supplementary.pdf]

Supplementary Material

We evaluated the performance of GTsurvival and other methods in Experiment 1-3. Even with varying parameters, GTsurvival outperformed other algorithms in terms of accuracy. Supplementary Figure S1-S4 are designed for Experiment 1. We observed the performance of GTsurvival in a variety of sample sizes $n = 200/400$ and feature counts $p = 50/100/500$. The results of Supplementary Figure S1-S4 indicated that GTsurvival can apply to datasets with non-linear relationship. Supplementary Figure S5-S10 are used to observe the generalization capability of GTsurvival in Experiment 2. GTsurvival performed best even under the different K groups. As for Experiment 3, GTsurvival obtained lower MSE and MAE than other methods in Supplementary Figure S11-S16. Supplementary Table 1-2 presented the MSE and MAE results of several algorithms used in Experiment 2-3. The results indicated the advantage of GTsurvival in predicting survival in complex censored data.

1. SUPPLEMENTARY FIGURE

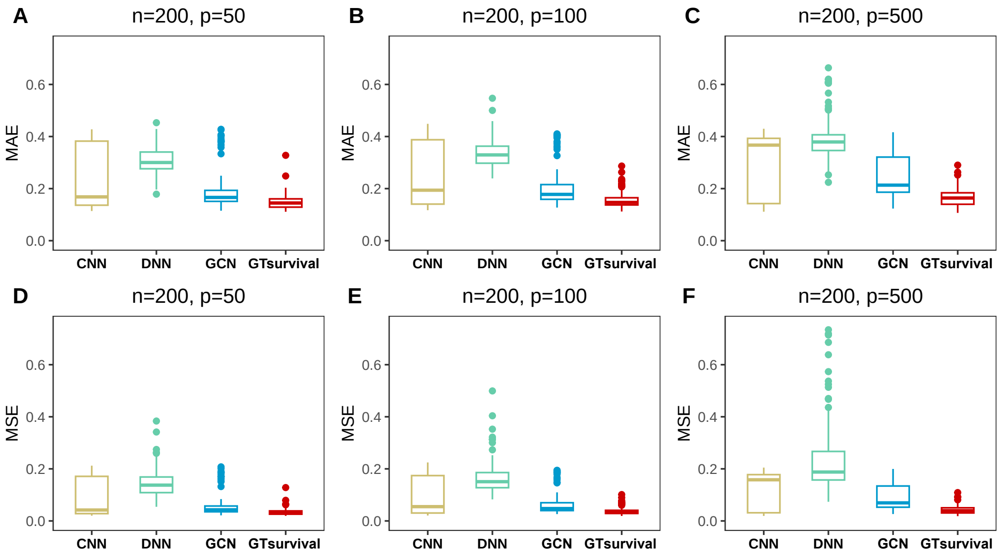

Figure S1. Box plots of MAE and MSE metrics in Experiment 1. The experiment was conducted with sample size $n = 200$ and feature counts $p = 50/100/500$. The RMST estimated at one ($K = 1$) time point ($\tau_1 = 1$ years).

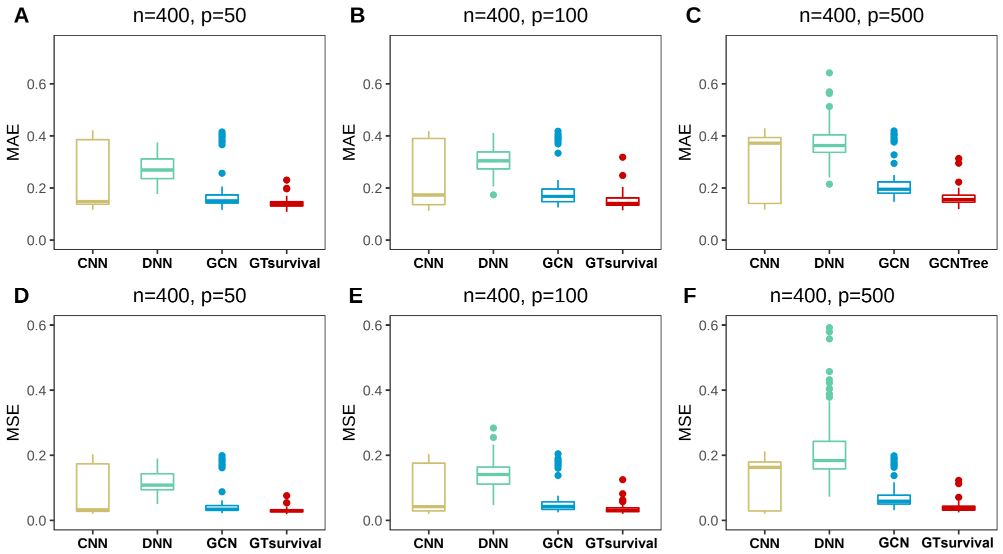

Figure S2. Box plots of MAE and MSE metrics in Experiment 1. The experiment was conducted with sample size $n = 400$ and feature counts $p = 50/100/500$. The RMST estimated at one ($K = 1$) time point ($\tau_1 = 1$ years).

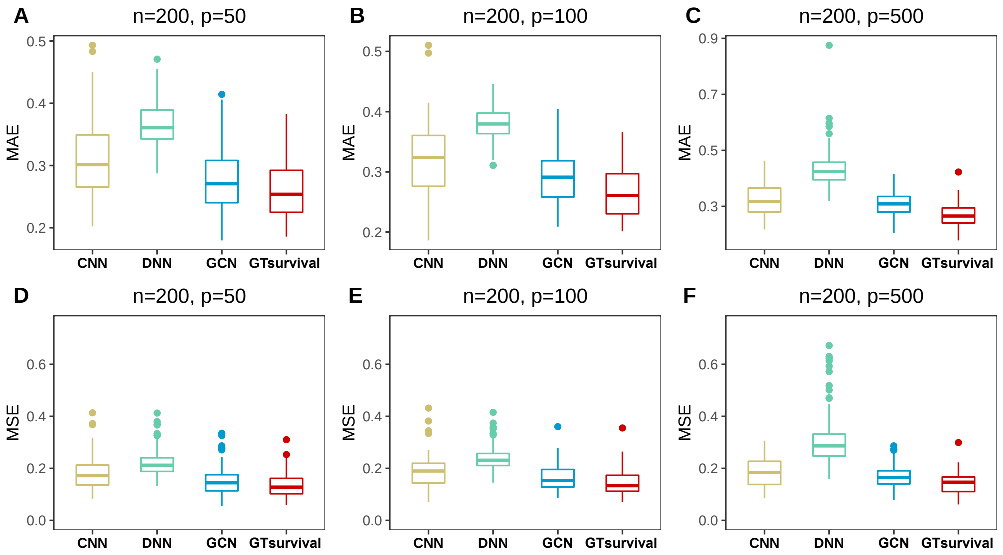

Figure S3. Box plots of MAE and MSE metrics in Experiment 1. The experiment was conducted with sample size $n = 200$ and feature counts $p = 50/100/500$. The RMST estimated at five ($K = 5$) time points ($\tau_1 = 1$, $\tau_2 = 3$, $\tau_3 = 5$, $\tau_4 = 7$, $\tau_5 = 9$ years).

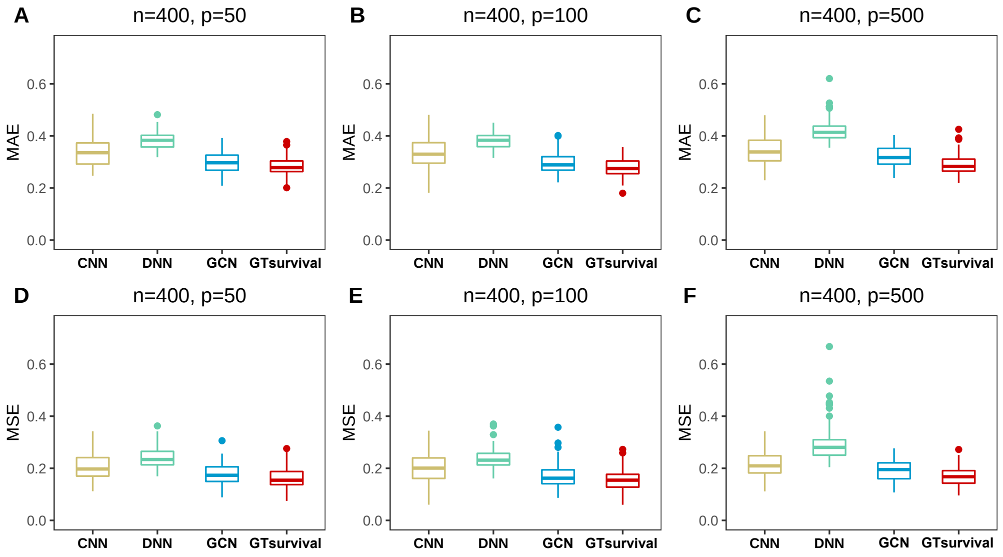

Figure S4. Box plots of MAE and MSE metrics in Experiment 1. The experiment was conducted with sample size $n = 400$ and feature counts $p = 50/100/500$. The RMST estimated at five ($K = 5$) time points ($\tau_1 = 1$, $\tau_2 = 3$, $\tau_3 = 5$, $\tau_4 = 7$, $\tau_5 = 9$ years).

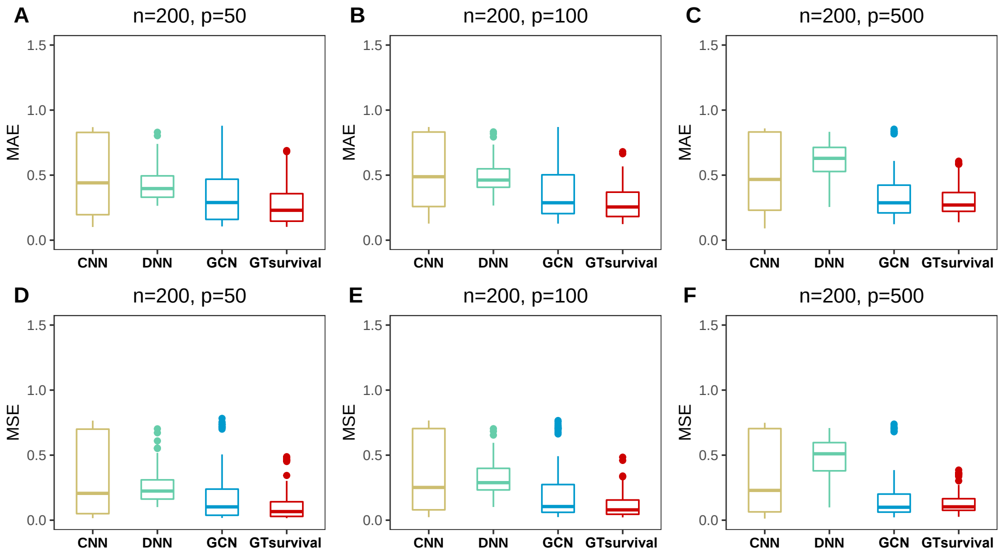

Figure S5. Box plots of MAE and MSE metrics in Experiment 2. The experiment was conducted with sample size $n = 200$ and feature counts $p = 50/100/500$. The RMST estimated at one ($K = 1$) time point ($\tau_1 = 1$ years).

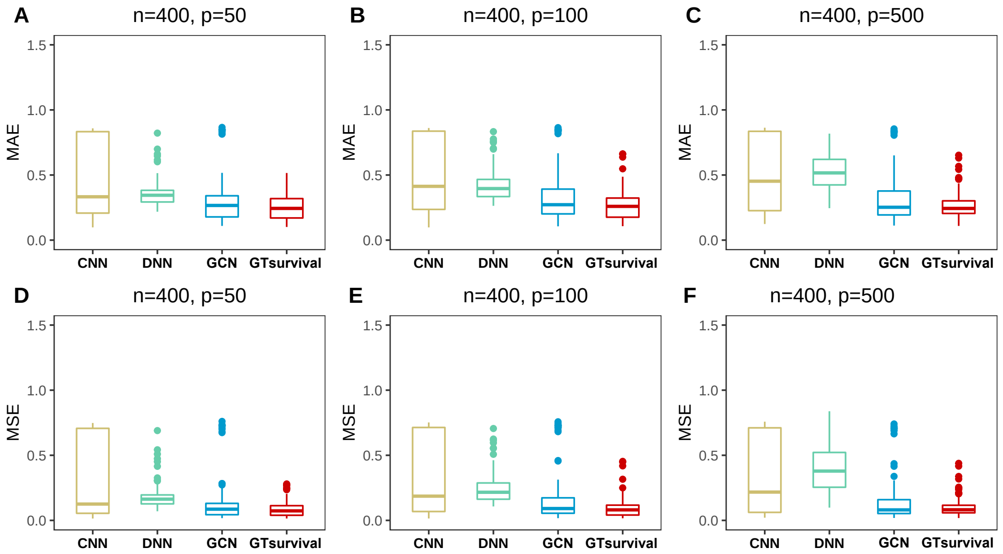

Figure S6. Box plots of MAE and MSE metrics in Experiment 2. The experiment was conducted with sample size $n = 400$ and feature counts $p = 50/100/500$. The RMST estimated at one ($K = 1$) time point ($\tau_1 = 1$ years).

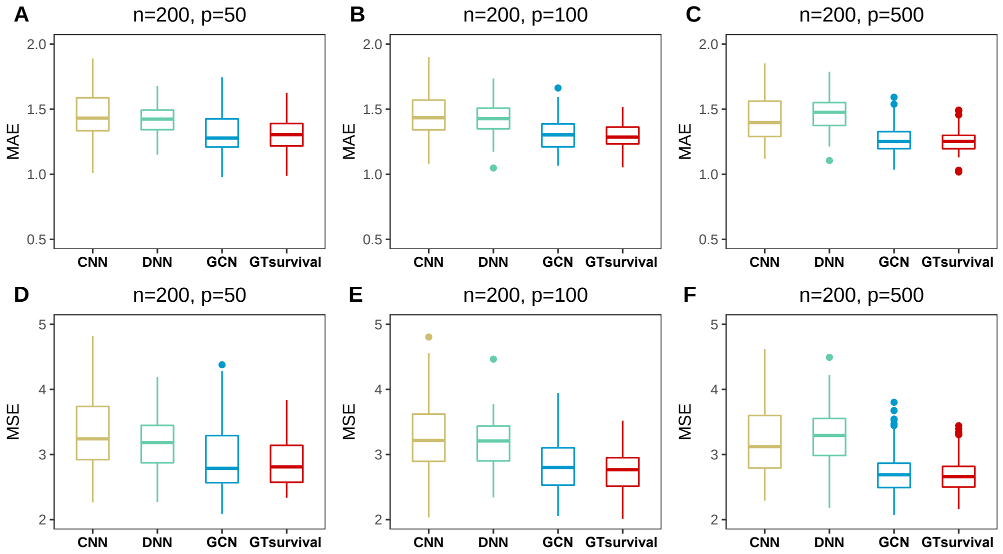

Figure S7. Box plots of MAE and MSE metrics in Experiment 2. The experiment was conducted with sample size $n = 200$ and feature counts $p = 50/100/500$. The RMST estimated at three ($K = 3$) time points ($\tau_1 = 1$, $\tau_2 = 3$, $\tau_3 = 5$ years).

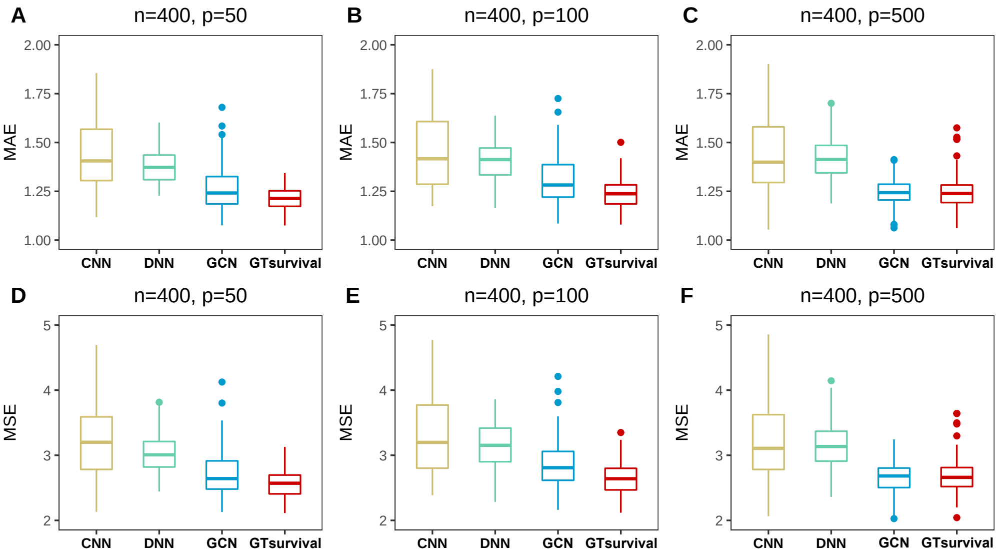

Figure S8. Box plots of MAE and MSE metrics in Experiment 2. The experiment was conducted with sample size $n = 400$ and feature counts $p = 50/100/500$. The RMST estimated at three ($K = 3$) time points ($\tau_1 = 1$, $\tau_2 = 3$, $\tau_3 = 5$ years).

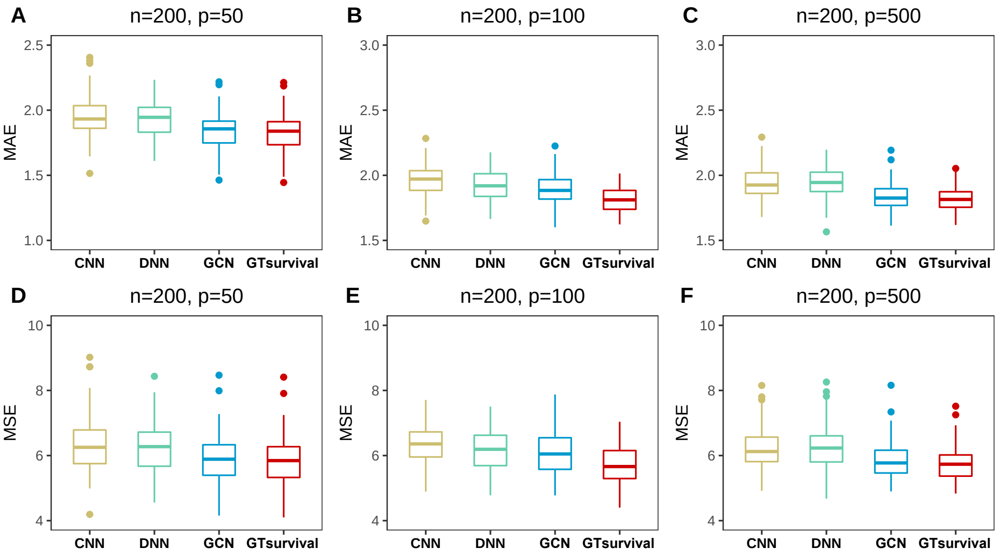

Figure S9. Box plots of MAE and MSE metrics in Experiment 2. The experiment was conducted with sample size $n = 200$ and feature counts $p = 50/100/500$. The RMST estimated at five ($K = 5$) time points ($\tau_1 = 1$, $\tau_2 = 3$, $\tau_3 = 5$, $\tau_4 = 7$, $\tau_5 = 9$ years).

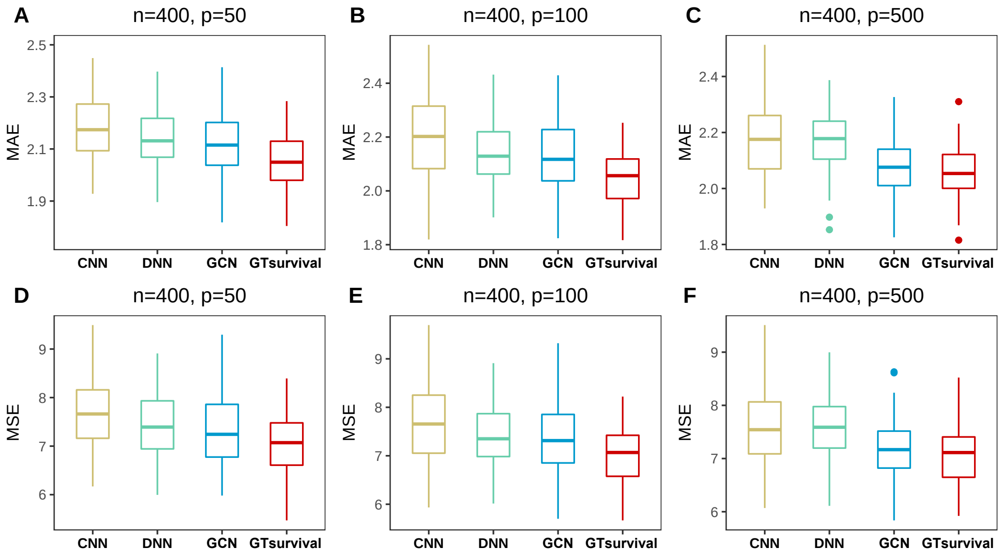

Figure S10. Box plots of MAE and MSE metrics in Experiment 2. The experiment was conducted with sample size $n = 400$ and feature counts $p = 50/100/500$. The RMST estimated at five ($K = 5$) time points ($\tau_1 = 1$, $\tau_2 = 3$, $\tau_3 = 5$, $\tau_4 = 7$, $\tau_5 = 9$ years).

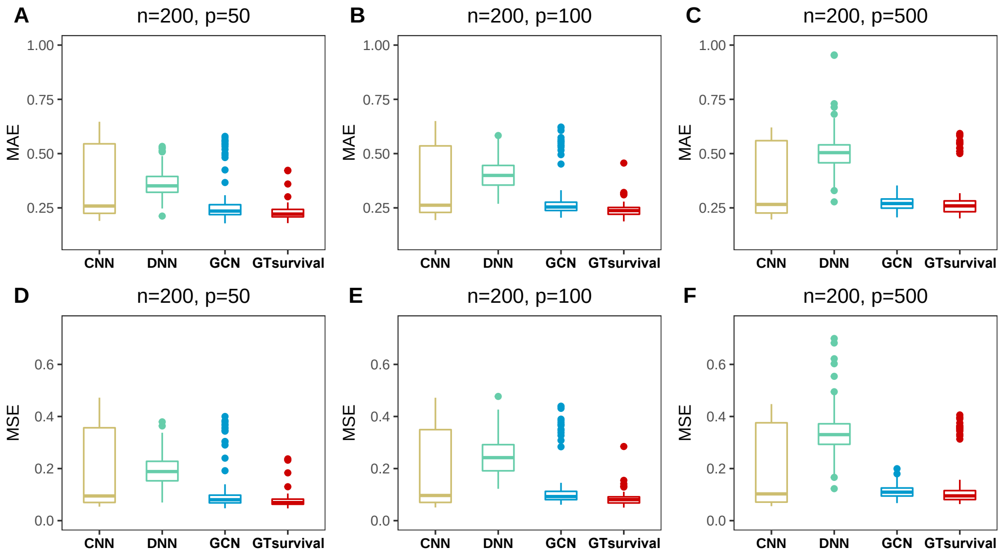

Figure S11. Box plots of MAE and MSE metrics in Experiment 3. The experiment was conducted with sample size $n = 200$ and feature counts $p = 50/100/500$. The RMST estimated at one ($K = 1$) time point ($\tau_1 = 1$ years).

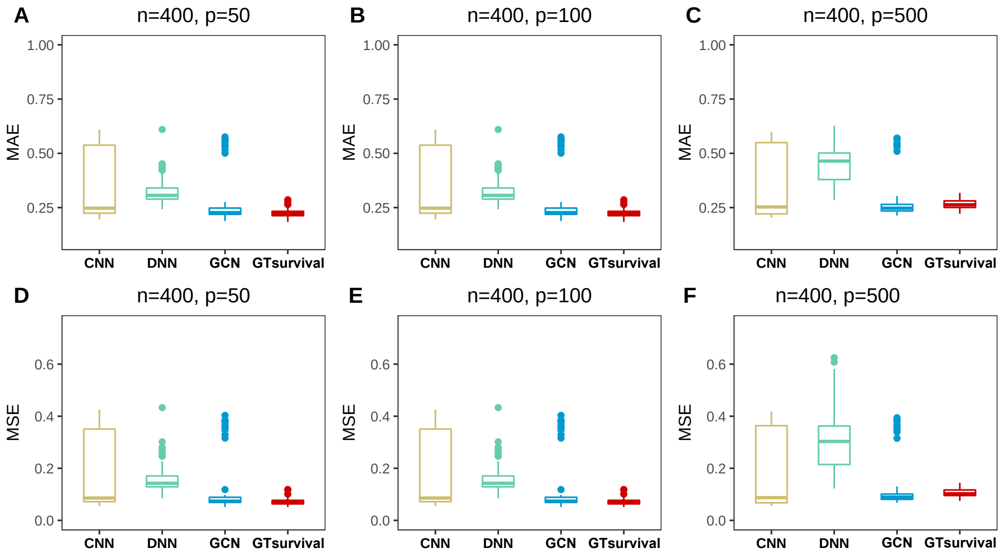

Figure S12. Box plots of MAE and MSE metrics in Experiment 3. The experiment was conducted with sample size $n = 400$ and feature counts $p = 50/100/500$. The RMST estimated at one ($K = 1$) time point ($\tau_1 = 1$ years).

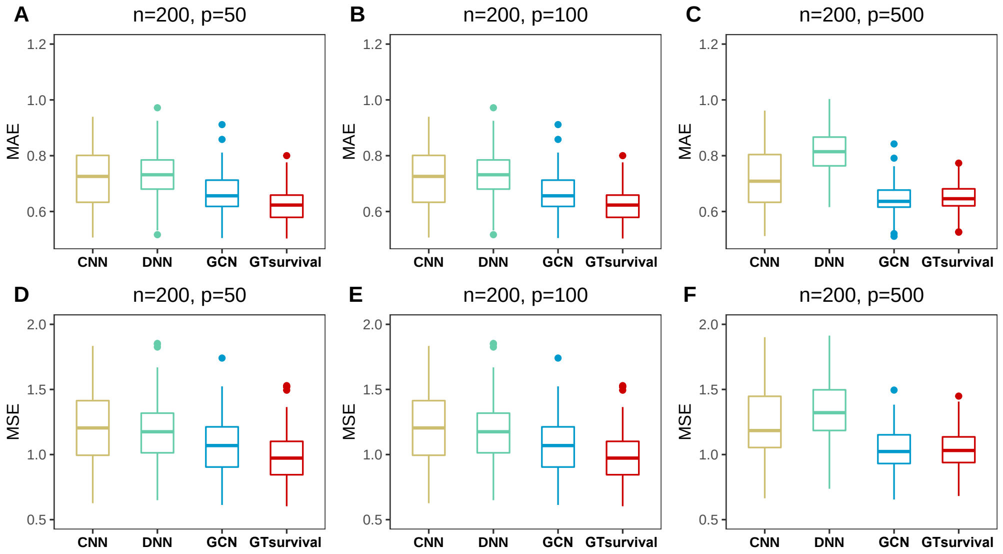

Figure S13. Box plots of MAE and MSE metrics in Experiment 3. The experiment was conducted with sample size $n = 200$ and feature counts $p = 50/100/500$. The RMST estimated at three ($K = 3$) time points ($\tau_1 = 1$, $\tau_2 = 3$, $\tau_3 = 5$ years).

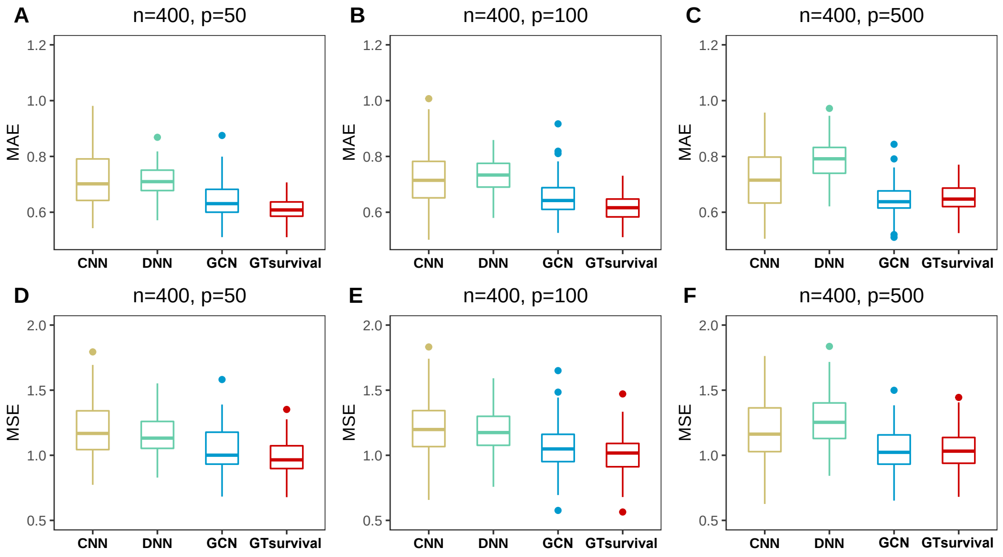

Figure S14. Box plots of MAE and MSE metrics in Experiment 3. The experiment was conducted with sample size $n = 400$ and feature counts $p = 50/100/500$. The RMST estimated at three ($K = 3$) time points ($\tau_1 = 1$, $\tau_2 = 3$, $\tau_3 = 5$ years).

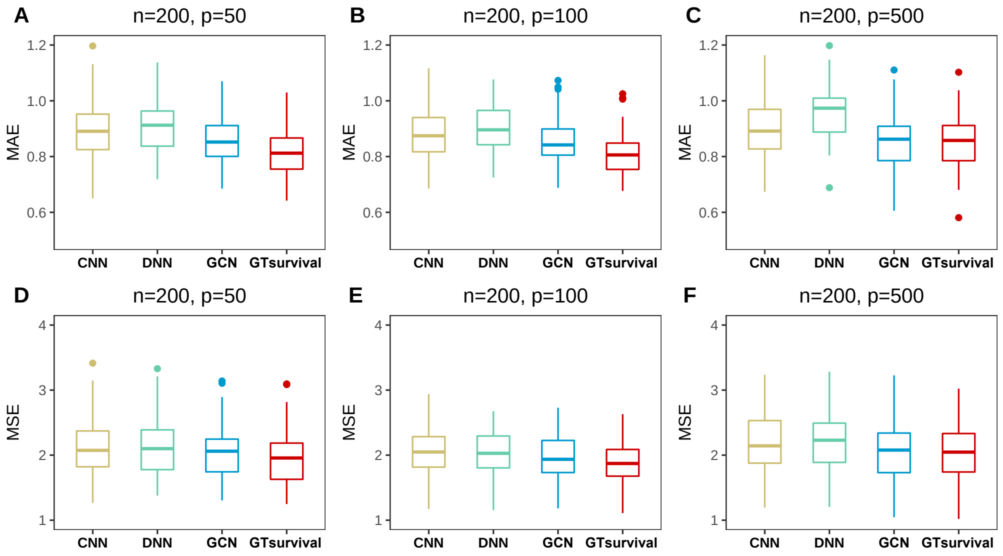

Figure S15. Box plots of MAE and MSE metrics in Experiment 3. The experiment was conducted with sample size $n = 200$ and feature counts $p = 50/100/500$. The RMST estimated at five ($K = 5$) time points ($\tau_1 = 1$, $\tau_2 = 3$, $\tau_3 = 5$, $\tau_4 = 7$, $\tau_5 = 9$ years).

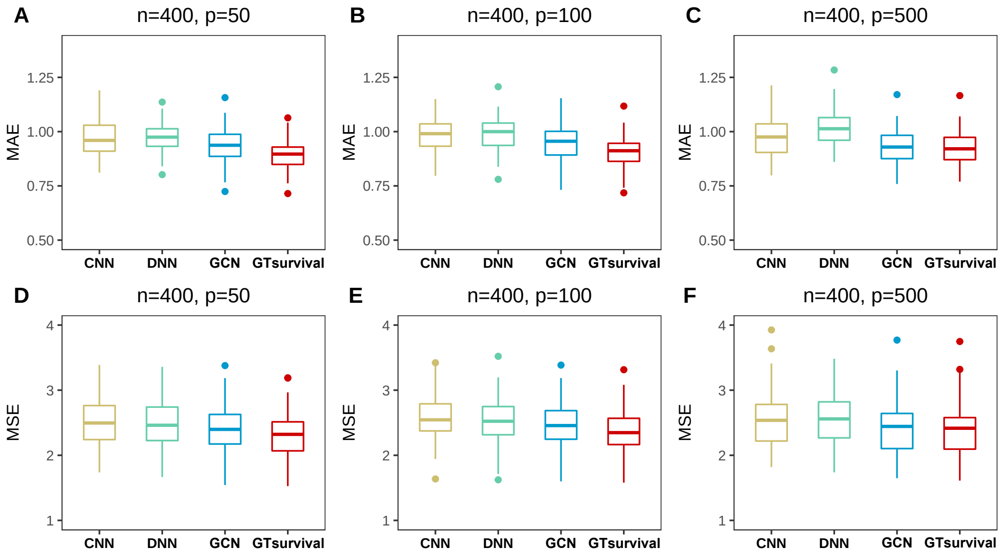

Figure S16. Box plots of MAE and MSE metrics in Experiment 3. The experiment was conducted with sample size $n = 400$ and feature counts $p = 50/100/500$. The RMST estimated at five ($K = 5$) time points ($\tau_1 = 1$, $\tau_2 = 3$, $\tau_3 = 5$, $\tau_4 = 7$, $\tau_5 = 9$ years)

2. SUPPLEMENTARY TABLE

Table S1. Simulation experiments on GTsurvival and the other three methods in Experiment 2. The sample sizes were set as $n = 200/400$, and feature counts were set as $p = 50/100/500$. The RMST was estimated at K time points, where $K \in \{1, 3, 5\}$. The specific observation times $\tau_1, \tau_2, \ldots, \tau_K$ were selected as follows: $\{1\}$, $\{1, 3, 5\}$, and $\{1, 3, 5, 7, 9\}$ years. Evaluation metrics are MSE and MAE, with smaller values corresponding to better prediction performance.The best performance is highlighted in bold, while the second-best result is underlined.

		MAE			MSE		
				$K = 1$			
	Method	$p = 50$	$p = 100$	$p = 500$	$p = 50$	$p = 100$	$p = 500$
$n = 200$	DNN	0.430(0.228)	0.490(0.288)	0.619(0.298)	0.260(0.146)	0.323(0.166)	0.485(0.311)
	CNN	0.494(0.103)	0.534(0.092)	0.528(0.118)	0.343(0.087)	0.380(0.098)	0.378(0.140)
	GCN	0.366(0.147)	0.391(0.129)	0.363(0.126)	0.210(0.074)	0.226(0.089)	0.195(0.062)
	GTsurvival	**0.273(0.122)**	**0.287(0.111)**	**0.306(0.089)**	**0.110(0.061)**	**0.113(0.062)**	**0.131(0.052)**
$n = 400$	DNN	0.356(0.295)	0.423(0.299)	0.528(0.300)	0.181(0.111)	0.251(0.314)	0.385(0.315)
	CNN	0.481(0.055)	0.509(0.087)	0.521(0.140)	0.329(0.042)	0.359(0.081)	0.372(0.163)
	GCN	0.315(0.087)	0.355(0.094)	0.324(0.100)	0.155(0.042)	0.193(0.058)	0.161(0.055)
	GTsurvival	**0.254(0.083)**	**0.271(0.095)**	**0.269(0.063)**	**0.086(0.042)**	**0.099(0.046)**	**0.104(0.036)**
				$K = 3$			
$n = 200$	DNN	1.420(0.171)	1.421(0.156)	1.472(0.175)	3.178(0.536)	3.154(0.494)	3.297(0.500)
	CNN	1.458(0.104)	1.455(0.098)	1.437(0.112)	3.302(0.387)	3.255(0.336)	3.223(0.378)
	GCN	1.317(0.137)	1.310(0.118)	1.269(0.080)	2.888(0.456)	2.838(0.381)	2.732(0.227)
	GTsurvival	**1.312(0.103)**	**1.290(0.089)**	**1.255(0.063)**	**2.856(0.330)**	**2.760(0.313)**	**2.681(0.195)**
$n = 400$	DNN	1.375(0.181)	1.410(0.190)	1.419(0.206)	3.025(0.547)	3.157(0.615)	3.155(0.597)
	CNN	1.432(0.080)	1.457(0.092)	1.436(0.098)	3.216(0.258)	3.317(0.333)	3.240(0.338)
	GCN	1.269(0.096)	1.311(0.111)	1.250(0.057)	2.730(0.303)	2.871(0.337)	2.684(0.221)
	GTsurvival	**1.214(0.056)**	**1.236(0.061)**	**1.243(0.062)**	**2.565(0.192)**	**2.645(0.219)**	**2.660(0.212)**
				$K = 5$			
$n = 200$	DNN	1.924(0.134)	1.918(0.101)	1.947(0.114)	6.217(0.721)	6.146(0.570)	6.262(0.544)
	CNN	1.957(0.132)	1.964(0.111)	1.939(0.109)	6.347(0.727)	6.322(0.628)	6.234(0.521)
	GCN	1.839(0.117)	1.893(0.107)	1.835(0.090)	5.888(0.669)	6.050(0.637)	5.830(0.506)
	GTsurvival	**1.825(0.117)**	**1.810(0.098)**	**1.817(0.085)**	**5.832(0.117)**	**5.711(0.574)**	**5.752(0.475)**
$n = 400$	DNN	2.138(0.129)	2.141(0.149)	2.172(0.122)	7.412(0.688)	7.422(0.779)	7.555(0.674)
	CNN	2.191(0.106)	2.199(0.113)	2.174(0.103)	7.641(0.638)	7.676(0.676)	7.567(0.539)
	GCN	2.123(0.125)	2.129(0.133)	2.076(0.085)	7.348(0.711)	7.374(0.720)	7.150(0.505)
	GTsurvival	**2.055(0.103)**	**2.048(0.099)**	**2.058(0.082)**	**7.041(0.590)**	**7.004(0.601)**	**7.067(0.517)**

Table S2. Simulation experiments on GTsurvival and the other three methods in Experiment 3. The sample sizes were set as $n = 200/400$, and feature counts were set as $p = 50/100/500$. The RMST was estimated at K time points, where $K \in \{1, 3, 5\}$. The specific observation times $\tau_1, \tau_2, \ldots, \tau_K$ were selected as follows: $\{1\}$, $\{1, 3, 5\}$, and $\{1, 3, 5, 7, 9\}$ years. Evaluation metrics are MSE and MAE, with smaller values corresponding to better prediction performance.The best performance is highlighted in bold, while the second-best result is underlined.

		MAE			MSE		
		$K = 1$					
	Method	$p = 50$	$p = 100$	$p = 500$	$p = 50$	$p = 100$	$p = 500$
$n = 200$	DNN	0.365(0.161)	0.403(0.159)	0.508(0.167)	0.200(0.146)	0.244(0.146)	0.384(0.153)
	CNN	0.899(0.051)	0.367(0.064)	0.380(0.057)	0.193(0.050)	0.199(0.066)	0.213(0.057)
	GCN	0.278(0.025)	0.295(0.023)	0.289(0.029)	0.119(0.014)	0.132(0.018)	0.128(0.021)
	GTsurvival	**0.230(0.022)**	**0.240(0.020)**	**0.272(0.027)**	**0.077(0.014)**	**0.083(0.013)**	**0.112(0.018)**
$n = 400$	DNN	0.318(0.156)	0.364(0.161)	0.445(0.166)	0.155(0.141)	0.201(0.146)	0.301(0.151)
	CNN	0.351(0.034)	0.368(0.054)	0.376(0.086)	0.185(0.029)	0.199(0.055)	0.208(0.095)
	GCN	0.271(0.018)	0.287(0.011)	0.284(0.017)	0.115(0.011)	0.128(0.009)	0.123(0.011)
	GTsurvival	**0.223(0.013)**	**0.230(0.020)**	**0.265(0.020)**	**0.071(0.009)**	**0.075(0.001)**	**0.105(0.015)**
		$K = 3$					
$n = 200$	DNN	0.734(0.112)	0.733(0.101)	0.818(0.116)	1.193(0.273)	1.193(0.220)	1.352(0.280)
	CNN	0.723(0.070)	0.724(0.073)	0.728(0.068)	1.206(0.216)	1.206(0.253)	1.232(0.238)
	GCN	0.661(0.073)	0.663(0.220)	0.661(0.044)	1.084(0.211)	1.061(0.079)	1.090(0.158)
	GTsurvival	**0.623(0.056)**	**0.621(0.065)**	**0.660(0.041)**	**1.019(0.182)**	**0.992(0.207)**	**1.083(0.138)**
$n = 400$	DNN	0.712(0.098)	0.729(0.090)	0.788(0.112)	1.154(0.212)	1.181(0.179)	1.270(0.252)
	CNN	0.718(0.049)	0.726(0.063)	0.721(0.061)	1.189(0.145)	1.211(0.148)	1.200(0.199)
	GCN	0.644(0.055)	0.651(0.052)	**0.646(0.044)**	1.046(0.161)	1.062(0.139)	**1.041(0.157)**
	GTsurvival	**0.612(0.036)**	**0.615(0.046)**	0.651(0.043)	**0.991(0.126)**	**1.003(0.124)**	1.044(0.139)
		$K = 5$					
$n = 200$	DNN	0.906(0.089)	0.897(0.095)	0.959(0.097)	2.134(0.401)	2.048(0.342)	2.227(0.445)
	CNN	0.893(0.078)	0.883(0.078)	0.898(0.082)	2.142(0.368)	2.063(0.341)	2.178(0.437)
	GCN	0.863(0.078)	0.852(0.067)	0.856(0.088)	2.064(0.363)	1.979(0.347)	2.067(0.427)
	GTsurvival	**0.817(0.074)**	**0.806(0.067)**	**0.852(0.085)**	**1.973(0.361)**	**1.889(0.319)**	**2.049(0.420)**
$n = 400$	DNN	0.975(0.081)	0.989(0.076)	1.017(0.087)	2.481(0.356)	2.540(0.295)	2.570(0.379)
	CNN	0.972(0.063)	0.987(0.064)	0.969(0.072)	2.504(0.337)	2.573(0.305)	2.529(0.382)
	GCN	0.936(0.072)	0.947(0.066)	0.930(0.074)	2.415(0.319)	2.464(0.300)	2.410(0.358)
	GTsurvival	**0.892(0.060)**	**0.905(0.056)**	**0.922(0.071)**	**2.315(0.292)**	**2.373(0.275)**	**2.384(0.330)**

Table S3. Statistical test results of GTsurvival vs. Second-Top baseline methods

p-value Selection Threshold	Evaluation Metric	Observation Time K	Second-Top Baseline Method	Wilcoxon Signed-Rank Test p-value
$p < 0.02$	MAE	1	CNN	2.03×10^{-9}
$p < 0.02$	MAE	3	DNN	1.82×10^{-5}
$p < 0.02$	MAE	5	GCN	3.18×10^{-7}
$p < 0.02$	MSE	1	DNN	9.34×10^{-8}
$p < 0.02$	MSE	3	DNN	1.42×10^{-6}
$p < 0.02$	MSE	5	DNN	3.77×10^{-9}
$p < 0.005$	MAE	1	GCN	2.00×10^{-7}
$p < 0.005$	MAE	3	GCN	1.26×10^{-4}
$p < 0.005$	MAE	5	DNN	3.86×10^{-7}
$p < 0.005$	MSE	1	GCN	1.81×10^{-8}
$p < 0.005$	MSE	3	GCN	3.80×10^{-3}
$p < 0.005$	MSE	5	DNN	8.10×10^{-8}
$p < 0.002$	MAE	1	GCN	1.59×10^{-6}
$p < 0.002$	MAE	3	GCN	5.66×10^{-7}
$p < 0.002$	MAE	5	GCN	5.68×10^{-13}
$p < 0.002$	MSE	1	DNN	2.95×10^{-10}
$p < 0.002$	MSE	3	GCN	3.07×10^{-3}
$p < 0.002$	MSE	5	GCN	1.74×10^{-10}

Table S4. Comparison of predictive performance among different methods on the LGG dataset (smaller values of MAE and MSE indicate better performance)

Metrics	DNN	CNN	GCN	GTsurvival	FastPseudo
MAE	0.986(0.048)	1.062(0.166)	1.005(0.132)	**0.8608(0.050)**	1.001(0.135)
MSE	2.512(0.274)	2.873(0.537)	2.615(0.427)	**1.9485(0.256)**	2.309(0.556)

Table S5. Results of ablation experiments on ADNI and TCGA-LGG datasets

Dataset	Method	MAE	MSE
ADNI	GCNet	1.330(0.345)	4.896(2.413)
	NDF	1.578(0.337)	5.401(2.598)
	FastPseudo	1.718(0.481)	5.504(3.137)
	GTsurvival	**1.033(0.159)**	**2.416(0.942)**
TCGA-LGG	GCNet	1.005(0.132)	2.615(0.427)
	NDF	1.782(0.061)	5.589(0.444)
	FastPseudo	1.001(0.135)	2.309(0.556)
	GTsurvival	**0.860(0.050)**	**1.948(0.256)**

3. DETAILED PROCESS OF GRADIENT DERIVATION

A. 1 Complete Steps for Deriving Decision Node Gradients

Step 1: Decomposition of Gradient Transmission Chain The relationship between the global loss and the loss of individual samples is:

$$\mathcal{L}(\Theta, \Lambda) = \frac{1}{nK} \sum_{i=1}^{n} L_{m,i},$$

where $L_{m,i} = \sum_{k=1}^{K} \left(\hat{y}_{ik} - g_i^*(X_i, \tau_k; \Theta, \Lambda)\right)^2$ is the cumulative loss of the i-th sample. The gradient of the global loss with respect to the decision node feature mapping $f_e(X_i; \Theta)$ can be converted into the average of the gradients of individual sample losses:

$$\frac{\partial \mathcal{L}(\Theta, \Lambda)}{\partial f_e(X_i; \Theta)} = \frac{1}{nK} \cdot \frac{\partial L_{m,i}}{\partial f_e(X_i; \Theta)},$$

Combined with the chain rule, the gradient transmission chain is $L_{m,i} \to g_i^* \to p_l \to d_e \to f_e$, substituting which gives:

$$\frac{\partial \mathcal{L}}{\partial f_e(X_i; \Theta)} = \frac{1}{nK} \sum_{k=1}^{K} \left(\frac{\partial L_{m,i}}{\partial g_i^*} \cdot \sum_{l \in L_d} \left(\frac{\partial g_i^*}{\partial p_l} \cdot \frac{\partial p_l}{\partial f_e} \right) \right),$$

where L_d is the number of leaf nodes.

Step 2: Calculation of Gradients for Each Component 1. Gradient of the loss with respect to the predicted value:

$$\frac{\partial \mathcal{L}}{\partial g_i^*} = \frac{2}{nK} \left(g_i^* - \hat{y}_{ik}\right) = \frac{2}{nK} r_{ik},$$

where r_{ik} denotes the prediction residual of the i-th sample at the k-th time point.

2. Gradient of the predicted value with respect to the routing probability:

From $g_i^* = \sum_{l \in L_d} \lambda_{lk} p_l$, we obtain:

$$\frac{\partial g_i^*}{\partial p_l} = \lambda_{lk}.$$

3. Gradient of the routing probability with respect to the feature mapping:
Logarithmic transformation is introduced to simplify differentiation:

$$\frac{\partial p_l}{\partial f_e} = p_l \cdot \frac{\partial \log p_l}{\partial f_e},$$

combined with the routing probability $\log p_l = \sum_{e \in E_d} \left(I_{l \swarrow e} \log d_e + I_{e \searrow l} \log \bar{d}_e \right)$ and the sigmoid derivative property $d'_e = d_e \cdot \bar{d}_e$, we can obtain:

$$\frac{\partial \log p_l}{\partial f_e} = I_{l \swarrow e} \cdot \bar{d}_e - I_{e \searrow l} \cdot d_e.$$

The final derivative of the routing probability is:

$$\frac{\partial p_l}{\partial f_e} = p_l \cdot \left(I_{l \swarrow e} \cdot \bar{d}_e - I_{e \searrow l} \cdot d_e \right).$$

Step 3: Gradient Merging and Simplification Substitute the three component gradients from Step 2 into the chain rule expression from Step 1:

$$\frac{\partial \mathcal{L}}{\partial f_e(X_i; \Theta)} = \frac{1}{nK} \sum_{k=1}^{K} \left(\frac{2}{nK} r_{ik} \cdot \sum_{l \in L_d} \left(\lambda_{lk} \cdot p_l \cdot \left(I_{l \swarrow e} \cdot \bar{d}_e - I_{e \searrow l} \cdot d_e \right) \right) \right).$$

Split the inner sum by indicator functions into left ($\mathcal{L}_{e_l}$) and right ($\mathcal{L}_{e_r}$) subtrees of node e:

$$\sum_{l \in L_d} \lambda_{lk} p_l \left(I_{l \swarrow e} \cdot \bar{d}_e - I_{e \searrow l} \cdot d_e \right) = \bar{d}_e \sum_{l \in \mathcal{L}_{e_l}} \lambda_{lk} p_l - d_e \sum_{l \in \mathcal{L}_{e_r}} \lambda_{lk} p_l.$$

Using $A_{e_l}^{(k)} = \sum_{l \in \mathcal{L}_{e_l}} \lambda_{lk} p_l$ and $A_{e_r}^{(k)} = \sum_{l \in \mathcal{L}_{e_r}} \lambda_{lk} p_l$, substitute to get:

$$\bar{d}_e \cdot A_{e_l}^{(k)} - d_e \cdot A_{e_r}^{(k)}.$$

Factor out $d_e \cdot \bar{d}_e$:

$$\bar{d}_e \cdot A_{e_l}^{(k)} - d_e \cdot A_{e_r}^{(k)} = d_e \cdot \bar{d}_e \cdot \left(A_{e_r}^{(k)} - A_{e_l}^{(k)} \right).$$

Substitute back into the global gradient expression to obtain the final formula:

$$\frac{\partial \mathcal{L}(\Theta, \Lambda)}{\partial f_e(X_i; \Theta)} = \frac{2}{nK} \cdot d_e(X_i; \Theta) \cdot \bar{d}_e(X_i; \Theta) \cdot \sum_{k=1}^{K} \left(r_{ik} \cdot (A_{e_r}^{(k)} - A_{e_l}^{(k)}) \right).$$

where $r_{ik} = g_i^* - \hat{y}_{ik}$, $\bar{d}_e = 1 - d_e$, and $\mathcal{L}_{e_l}, \mathcal{L}_{e_r}$ are the leaf sets of node e's left/right subtrees.

B. 2 Complete Steps for Deriving Leaf Node Gradients

According to the chain rule, the gradient transmission path is $\mathcal{L} \to g_i^* \to \lambda_{lk}$, that is:

$$\frac{\partial \mathcal{L}}{\partial \lambda_{lk}} = \frac{\partial \mathcal{L}}{\partial g_i^*} \cdot \frac{\partial g_i^*}{\partial \lambda_{lk}}$$

Substituting $\frac{\partial \mathcal{L}}{\partial g_i^*} = \frac{2}{nK} r_{ik}$ (where $r_{ik} = g_i^* - \hat{y}_{ik}$) and $\frac{\partial g_i^*}{\partial \lambda_{lk}} = p_l$, the gradient formula for leaf node parameters is :

$$\frac{\partial \mathcal{L}(\Theta, \Lambda)}{\partial \lambda_{lk}} = \frac{2}{nK} \cdot r_{ik} \cdot p_l(X_i | \Theta).$$
